# PfSWIB, a potential chromatin regulator for *var* gene regulation and parasite development in *Plasmodium falciparum*

**DOI:** 10.1186/s13071-020-3918-5

**Published:** 2020-02-04

**Authors:** Wei-Feng Wang, Yi-Long Zhang

**Affiliations:** 1 Central Laboratory, Shanghai Tenth People’s Hospital, Tongji University, Shanghai, 200072 China; 2 Department of Tropical Diseases, Faculty of Naval Medicine, Second Military Medical University, Shanghai, 200433 China; 3 Institute for Infectious Diseases and Vaccine Development, Tongji University School of Medicine, 1239 Siping Road, Shanghai, 200092 China

**Keywords:** *Plasmodium falciparum*, Chromatin regulator, Clonal variation, *var* gene, Regulation

## Abstract

**Background:**

Various transcription factors are involved in the process of mutually exclusive expression and clonal variation of the *Plasmodium* multigene (*var*) family. Recent studies revealed that a *P. falciparum* SWI/SNF-related matrix-associated actin-dependent regulator of chromatin (*PfSWIB*) might trigger stage-specific programmed cell death (PCD), and was not only crucial for the survival and development of parasite, but also had profound effects on the parasite by interacting with other unknown proteins. However, it remains unclear whether *PfSIWB* is involved in transcriptional regulation of this virulence gene and its functional properties.

**Methods:**

A conditional knockdown system “*PfSWIB*-FKBP-LID” was introduced to the parasite clone 3D7, and an integrated parasite line “*PfSWIB*-HA-FKBP-LID” was obtained by drug cycling and clone screening. Growth curve analysis (GCA) was performed to investigate the growth and development of different parasite lines during 96 h *in vitro* culturing, by assessing parasitemia. Finally, we performed qPCR assays to detect *var* gene expression profiling in various comparison groups, as well as the mutually exclusive expression pattern of the *var* genes within a single 48 h life-cycle of *P. falciparum* in different parasite lines. In addition, RNA-seq was applied to analyze the *var* gene expression in different lines.

**Results:**

GCA revealed that conditional knockdown of *PfSWIB* could interfere with the growth and development of *P. falciparum*. The parasitemia of *PfSWIB∆* showed a significant decline at 96 h during *in vitro* culture compared with the *PfSWIB* and 3D7 lines (*P* < 0.0001). qPCR and RNA-seq analysis confirmed that depletion of *PfSWIB* not only silences *upsA*, *upsC* and partial *upsB var* genes, as well as removes the silencing of partial *upsB var* genes at the ring stage in *PfSWIB∆* line, but also leads to aberrant expression of *upsA* and partial *upsB/upsC var* genes at the mature stage of *P. falciparum*, during a single 48-h life-cycle.

**Conclusions:**

We demonstrated that *PfSWIB* was involved in the process of clonal variation in *var* gene expression, and crucial for the survival and development of *Plasmodium* parasite. These findings could provide better understanding of the mechanism and function of *PfSWIB* contributing to the pathogenesis in malaria parasites.
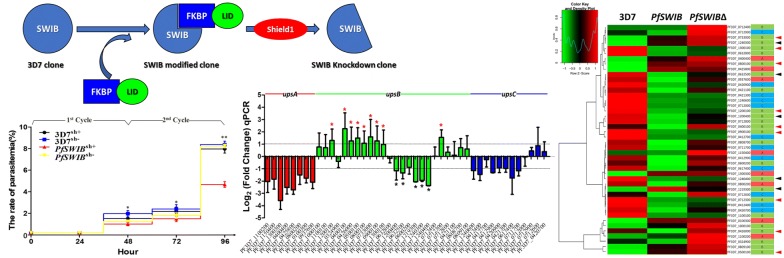

## Background

*Plasmodium falciparum*, which causes malignant malaria such as cerebral malaria (CM) or pregnancy-associated malaria (PAM) [1, 2], is the cause of death to 435,000 individuals annually [3]. *Plasmodium falciparum* erythrocyte membrane protein 1 (PfEMP1), which is encoded by the *var* gene family comprising approximately 60 members, is the major virulence factor involved in the antigenic variation and clinical pathogenicity of falciparum malaria [4, 5]. It is remarkable that the expression of *var* family members is mutually exclusive. For each parasite at a time, only one or a few *var* genes are expressed, while the remaining members are silenced [6, 7]. In each generation, *P. falciparum* is able to express different *var* genes, and the switches in *var* gene expression lead to antigenic variation of PfEMP1, which results in immune evasion and chronic infection [8–11]. This process has proven to be mediated by epigenetic mechanisms, including chromatin modification, nuclear architecture and gene relocation [12, 13].

It has been shown that the single active *var* gene is enriched in euchromatic modifications, such as histone 3 lysine 9 acetylation (H3K9ac) and histone 3 lysine 4 trimethylation (H3K4me3), particularly near the transcriptional start site (TSS) [14], while the silent *var* genes are usually enriched in the heterochromatin marker (histone 3 lysine9 trimethylation, H3K9me3) in the 5’UTR or the coding sequence [14–17]. In this process, histone modifying enzymes such as *PfMYST*, *PfGCN5*, *PfSIR2* and *PfSET*, have been considered important for epigenetic control of reversible histone modifications, according to previous studies [12, 13]. *PfMYST*, a putative histone acetyltransferases (HATs) in *P. falciparum*, is capable of acetylating multiple lysines on H4 [18, 19], while an orthologue of the yeast HAT *PfGCN5* influences acetylation of H3K9 and K14, both are essential marks of gene activation [18, 20]. Two paralogues of the class III HDAC (histone deacetylases) Sir2, play key roles in maintaining heterochromatin and mutually exclusive *var* gene expression [15, 21, 22]. PfSir2A deacetylates H3K9ac, H3K14ac and H4K16ac [23], and is more important in silencing subtelomeric *var* genes such as *upsA-*, *upsE-* and *upsC*-subtype *vars*. In contrast, PfSir2B silences *upsB vars* [22]. Furthermore, a total of ten *P. falciparum* histone lysine methyltransferases (HKMTs) belonging to the SET domain family have been found in *P. falciparu*m [18, 24, 25]. *PfSET10* localizes to a specialized region at the nuclear periphery with the active *vars* such as H3K4me4 transferase [26].

*Plasmodium falciparum* variant-silencing SET gene (PfSETvs) controls the H3K36me3 on *var* genes (silencing *var* genes) [27]. Moreover, subnuclear architecture contributes to the regulation of *var* gene expression [13]. *var* genes are by default silent since they are located at the nuclear periphery [28]. However, active *var* promoters seem to occupy a privileged expression site of this region, making it permissive to transcribe [21]. PF11_0091, an ApiAP2 member, binds a conserved motif in *var* introns, mediates episomal anchoring towards the nuclear periphery, and recruiting an actin protein complex and polymerized nuclear actin de-represses silent *var* genes [29].

On the other hand, various trans factors are intimately involved in regulating chromatin structure and gene activity. PfAlbas contribute to heterochromatin structure and regulation of *var* gene expression [30]. PfSIP2, another ApiAP2 member, is involved in the recruitment and organization of subtelomeric heterochromatin through binding to SPE2 [31], and induces a comprehensive silencing of a *var* promoter. Particularly, four major classes of ATP-dependent chromatin remodelers had been found in *P. falciparum*, facilitating the spatial and temporal remodeling of chromatin [13]. All these remodeler families consist of a SWI2/SNF2-family ATPase subunit featuring an ATPase domain [32, 33], which includes an HSA (helicase-SANT), a post-HSA, and a C-terminal bromodomain. Some other conserved subunits carry extra conserved domains, such as hBAF155/170 (SANT, SWIRM), hBAF60 (SWIB) and human polybromo (multiple bromodomains). BAF60b is generally known as SWI-B or SWIB, an alternative form of the SWI/SNF complex (Complex B) [34], while human BAF60a protein of SWI/SNF complex is responsible for facilitating cell cycle halting and has the ability to tune the balance between repair and apoptosis induction [35–37]. Recently, a SWI/SNF-related matrix-associated actin-dependent regulator of chromatin, *PfSWIB* (GenBank: PF3D7_0611400), which is characterized by its SWIB/MDM2 domain (SWI complex, BAF60b domain), has been identified in *P. falciparum*. *PfSWIB* might trigger stage-specific PCD by which transient nuclear localization causes removal of these parasites [38]. Further studies revealed that *PfSWIB* was not only crucial for the survival and growth of the parasite, but also had profound effects on the parasite by interacting with other unknown proteins [38–40]. However, it remains unclear whether *PfSWIB* is involved in transcriptional regulation of virulence gene, especially *vars*, and its functional properties.

To address the molecular mechanism of *PfSWIB*, we propose a new conditional knockdown system to study the function and mechanism of chromatin remodeler *PfSWIB* in regulating *var* gene expression. We found that the dysfunction of *PfSWIB* might lead to a significant downregulation of *upsA*, *upsC* and partial *upsB var* genes at the ring stage in a *PfSWIB*-knockdown line, as well as the aberrant expression of certain *var* genes at the mature stage in a single 48-h life-cycle of *P. falciparum*, which was characterized by the reversion of transcriptional downregulation of *upsA* and partial *upsB/upsC var* genes, suggesting that *PfSWIB* might play a key role on *var* gene regulating. In addition, *PfSWIB* knockdown could affect the growth and development of parasite. This study provides a better understanding of the regulatory function of chromatin remodeling complexes (CRCs) in regulating the clonal variation of *var* genes, and an essential insight into the regulation of the major virulence gene family contributing to the pathogenesis in malaria parasites.

## Methods

### Parasite culture

*Plasmodium falciparum* lines 3D7, *PfSWIB* and *PfSWIB*∆ were first thawed and established for continuous cultivation *in vitro* according to standard procedures [41]. The cultivation was as follows: thawed isolates were cultured in RPMI 1640 medium (Invitrogen/Thermo Fisher Scientific, Carlsbad, CA, USA) containing 25 mM Hepes, 2 mM l-glutamine, 0.1 mM hypoxanthine (Sigma-Aldrich, Shanghai, China), 20 mg/ml gentamicin (Sigma-Aldrich, Shanghai, China), 0.5% Albumax II (Invitrogen/Thermo Fisher Scientific) and 2% human serum (type AB+; Changhai Hospital, The Second Military Medical University, Shanghai, China). Cultures were grown in media with type O+ erythrocytes at a 3% hematocrit under 2% O_2_, 5.5% CO_2_, 92.5% N_2_ at 37 °C. To obtain tightly synchronized ring stage parasites, a standard percoll/sorbitol synchronization method was performed [42, 43]. In brief, schizonts were isolated over a 40 and 60% percoll cushion and allowed to invade fresh RBCs for 0 to 6 h. The resulting rings were subjected to 5% sorbitol treatment for 5 min at 37 °C. This process was repeated twice. The culture was washed and re-suspended in complete medium containing 2% RBC after the final sorbitol treatment.

### DNA isolation and transfection efficiency detection of *PfSWIB* modified locus in 3D7 and transfected lines

All transfected parasite lines originated from the 3D7 clone. DNA extraction was performed using the QIAamp DNA Mini Kit (Qiagen, Hilden, Germany). The high fidelity KOD-plus-Neo enzyme (Toyobo, Osaka, Japan) was used for PCR amplification to detect the endogenous *PfSWIB* locus or integration of the *PfSWIB* locus (*PfSWIB*-HA-FKBP-LID). PCR amplification reaction conditions are as follows: 95 °C for 3 min, followed by 32 cycles of 95 °C for 30 s, 50 °C for 30 s, 62 °C for 1 min, and a final extension cycle of 65 °C for 10 min. The specific primers used for PCR are listed in Additional file 1: Table S1.

### Construction of recombination fusion system *PfSWIB*-HA-FKBP-LID

**Fig. 1:**
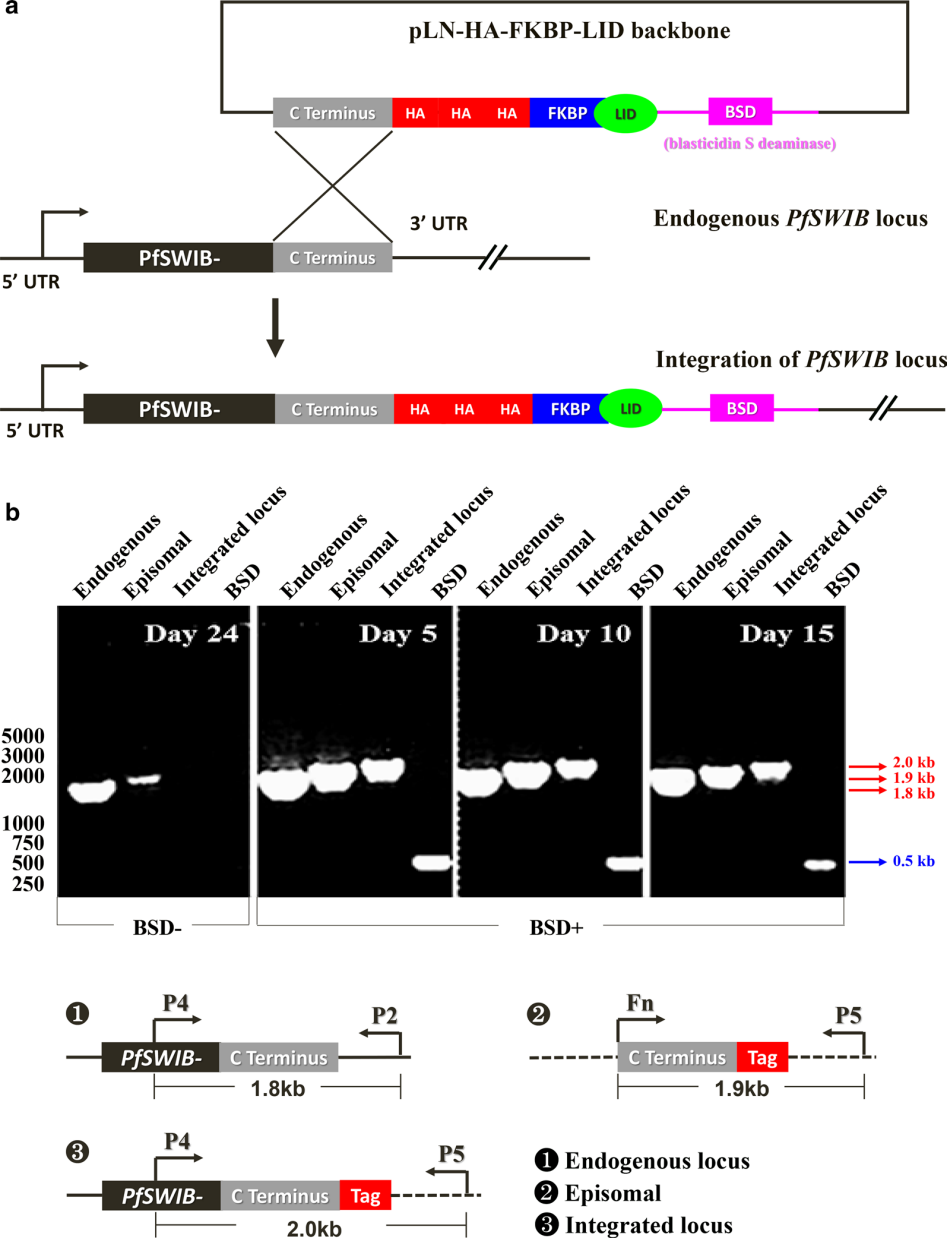
Transfection and drug screening of the *PfSWIB* fusion expression system. **a** Schematic illustration of conditional *PfSWIB* knockdown strategy in the 3D7 clone. The pLN-HA-FKBP-LID construct was derived from plasmid pLN-ENR-GFP [44], which harbored an antibiotic selection marker (*bsd* for Blasticidin S resistance, pink). HA tags (red) and FKBP-LID tag (blue and green) were ligated towards the 1.5 kb C terminus of *PfSWIB*. **b** Drug screening of the *PfSWIB* fusion expression system. The integration events and expression of *PfSWIB* modified locus were identified by PCR. ‘BSD+’ and ‘BSD-’ indicate that the transfected parasites were cultured in the presence or absence of BSD. Day 24: *in vitro* culturing in the absence of BSD for 24 days. Day 5, 10 and 15: *in vitro* culturing in the presence of BSD for 5, 10 and 15 days, respectively. The details of specific primers used for detecting the endogenous, episomal and integrated *PfSWIB* locus are provided in “Methods”

pLN-ENR-GFP [44] (GenBank: DQ813653.3) was modified by deleting the whole *pfenr-gfp* cassette, which was then used to clone various DNA fragments in *Apa*I/*Bam*HI multicloning site. For the pLN-HA-FKBP-LID construct (6900 bp), the calmodulin promoter (5’ CaM fragment) was initially substituted by a 1.5 kb C terminus of *PfSWIB* in the *Apa*I/ASCI multicloning site. Three duplicates of HA tag (YPYDVPDYA) and FKBP-LID tag were ligated towards the C terminus of *PfSWIB via* a linker sequence (AAAAVDAAAA). The resulting vector pLN-*PfSWIB*-HA-FKBP-LID was used to transfect the 3D7 clone. The HA tag consists of three tandem repeat sequences of influenza hemagglutinin, whereas the FKBP-LID tag is comprised of the FK506- and rapamycin-binding protein and a 19-amino-acid degron fused towards the C terminus of FKBP (Fig. 1a) [45].

### Transgenosis and drug screening

Transfections were performed on synchronized ring-stage parasites of 3D7 in a 2 mm BioRad Gene Pulser® cuvettes (Bio-Rad Laboratories, Hercules, CA 94547, USA). A minimum of 100 μg recombinated plasmid DNA was initially purified and used for electroporation using the Bio-Rad GenePulse Xcell™ electroporator (BioRad Laboratories, Hercules, CA 94547, USA), at 310 V, with a resistance of 950 μF and a transfection time of less than 15 ms. Positive BSD drug selection at the final concentration of 2 nM, was subsequently applied and maintained after the first growth cycle. Those parasites that adopted the pLN-*PfSWIB*-HA-FKBP-LID construct carrying the *bsd* gene would offer a level of resistance to the drug. The transfected parasites were subjected to *in vitro* culturing for 25 to 40 days and subsequent BSD drug cycling to select stable single-crossover parasites. The initial obtained transfected parasite line was a mixture of the episomal and integrated form. Therefore, a “3 + 2” mode of drug screening process was subsequently introduced to enrich the integrated lines. In this process, the original transfected parasites were first cultured *in vitro* in the absence of BSD for 3 weeks, followed by 2 weeks of culture with BSD (2.5 µg/ml), therefore integrated lines of *PfSWIB*-HA-FKBP-LID could possibly be further sorted after 2–3 rounds of drug cycling (Fig. 1b). After two cycles of BSD “off” (3 weeks) and BSD “on” (2 weeks), PCR was applied to detect the integration events of *PfSWIB*-HA-FKBP-LID in transfected parasites lines by using specific primers (Additional file 1: Table S1). Primers P2, P4 and P5 are specific to 3’UTR of endogenous *PfSWIB*, the upstream sequence of *PfSWIB* C-terminus and downstream plasmid sequence of HA-FKBP-LID fusion expression system, respectively, while Fn is the forward primer used for PCR amplification of *PfSWIB* C-terminus.

### Screening of integrated parasites clones

In order to obtain purified integrated parasites clones, we utilized a clone screening assay by serial dilution of parasites in 96-well plates, as detailed previously [46]. Two stable transfections of *PfSWIB*-FKBP-LID integrated parasites clones, *PfSWIB*-C2 and *PfSWIB*-B10 were obtained. The integration events were detected on screened clones after extended the culture to 24-well plates. The primers used for testing the clones are described above (see also Additional file 1: Table S1). PCR amplification reaction conditions were as follows: 94 °C for 5 min, followed by 32 cycles of 94 °C for 30 s, 54 °C for 30 s, 65 °C for 2 min, and a final extension cycle of 65 °C for 10 min.

### Western blotting

To analyze the expression of the *PfSWIB* fusion system in integrated parasites lines, total parasite extracts were prepared by treatment with 0.15% saponin and re-suspended in SDS-loading buffer (Bio-Rad), then separated on a 12% SDS-PAGE gel (Bio-Rad) and subjected to western blot analysis. Total proteins from the 3D7 clone were used as controls. An antibody to aldolase (1:1000 dilution; Roche, Indianapolis, USA) was used as a positive control. Rabbit anti-HA (1:1000 dilution; Abcam, Cambridge, UK) was used to identify *PfSWIB* fusion proteins in different parasite lines. An enhanced chemiluminescence (ECL) western blotting kit (GE Healthcare, Uppsala, Sweden) was used to develop western blots. The theoretical molecular weight of endogenous *PfSWIB*, HA tag and HA-FKBP-LID tag is 92kD, 3.5kD and 22kD, respectively. The gray-level image analysis procedure was implemented with ImageJ software. Statistical significance was determined (*P < 0.05, **P < 0.01).

### Growth curve analysis

Synchronized ring parasites (0–6 h after re-invasion) of the *PfSWIB* integrated clone (*PfSWIB*-HA-FKBP-LID) and the 3D7 clone were collected and diluted to 0.2% parasitemia. Each diluted parasite clone was divided into two wells of parasite cultures in the 24-well plate (50 µl RBC pellets/1 ml medium). Shield1 (0.5 mM (diluted in ethanol); Clontech, Mountain View, USA) was added into one of the two wells (final concentration: 0.5 μM). Consequently, four parasite lines including *PfSWIB*, *PfSWIB*Δ, 3D7 (shield1 induced) and 3D7^sh−^ (shield1 not induced) were subject to *in vitro* culture for 4–5 days. The parasitemia of each parasite line was counted every 12 h up to 96 h and repeated twice. The rate of parasitemia (%) of different lines were compared statistically by a one-way ANOVA followed by Bonferroni’s multiple comparison test (*P < 0.05, **P < 0.01). Growth curve analysis was performed in triplicate.

### RNA extraction and quantitative real-time PCR

For the transcription level assay of individual *var* genes, we used the *var* primer pairs published elsewhere [47] and listed in Additional file 2: Table S2. qPCR assays were performed as described previously [10]. In brief, total RNA of synchronous parasite culture was extracted using TRIzol reagent (Invitrogen/Thermo Fisher Scientific, Carlsbad, CA, USA) [48]. cDNA was prepared in accordance with the manufacturer’s recommendations (Thermofisher Scientific, Carlsbad, CA, USA). The fold change between the experimental and reference groups was calculated as 2^−ΔΔCq^. qPCR was performed on a QuantStudio 6 Flex Real-Time PCR System (Applied Biosystems, Singapore) using a program of 1 cycle of 15 s at 95 °C and 40 cycles of 30 s at 95 °C, 30 s at 54 °C, 30 s at 60 °C. Seryl-tRNA synthetase was applied as an endogenous control gene and to normalize the transcriptional level of each *var* gene. For the qPCR data, statistical significance was determined with the two-tailed Student’s t-test (*P < 0.05, **P < 0.01).

### RNA-seq data analysis

RNA-seq was used to screen differentially expressed genes in three parasite lines and was carried out in duplicate. The reference genome and transcript information (*P. falciparum* 3D7) were obtained from PlasmoDB (http://plasmodb.org/). The RNA-seq aligner STAR v2.5.3a [49] was used to create a genome catalog, based on two data files including a genome sequence fasta file and GFF3 annotation file (PlasmoDB-34_Pfalciparum3D7_Genome.fasta, PlasmoDB-34_Pfalciparum3D7.gff). FastQC v0.11.5 was subsequently used to analyze the quality of the RNA-seq reads. The qualified reads were subsequently mapped and aligned to the reference genome. Reads with a minimum mapping quality of 20 (− q 20) were further screened out by using SAM tools. Heatmaps of gene expression profiles in different lines were created by using the R package *edgeR* [50]. Hierarchical clustering was performed by using one minus Pearson’s correlation metric and average linkage method. Line plots were generated with *ggplot2* [51]. In order to carry out an analysis of variance (ANOVA)-like differential gene expression analysis, a statistical analysis of summarized read counts per genomic feature obtained from feature Counts function [52] was performed by using the R package *ALDEx2* [53, 54]. A fold change greater than 1.5 or lower than − 1.5, FDR < 0.05, and *P* < 0.05 were selected as filters for defining the sets of differentially expressed genes.

## Results

### Transfection and drug screening of the *PfSWIB* fusion expression system

Due to the low transfection proficiency after inserting the full-length *PfSWIB* gene, we subsequently selected a 1.5 kb C terminal sequence of *PfSWIB* and cloned this into the modified recombinant plasmids pLN-ENR-GFP forming fusion gene expression system pLN-*PfSWIB* (C-terminus)-HA-FKBP-LID. Through homologous recombination caused by a single crossover event, a transfected parasite clone known as *PfSWIB*-HA-FKBP-LID was obtained by transfecting the recombinant plasmid pLN-*PfSWIB* (C-terminus)-HA-FKBP-LID into the 3D7 strain, which was selected by Blasticidin S deaminase (BSD). The HA tag contains three tandem repeat sequences of influenza hemagglutinin, whereas FKBP-LID tag is comprised of the FK506- and rapamycin-binding protein and a 19-amino-acid degron (ligand-induced degradation domain) fused to the C terminus of FKBP (Fig. 1a) [45]. A drug cycling process was subsequently introduced to enrich the integrated lines. The integration events and expression of *PfSWIB*-HA-FKBP-LID fusion gene were first identified by PCR (Fig. 1b). The specific primers designed for detecting the transfection proficiency of the *PfSWIB* modified locus are listed in Additional file 1: Table S1. Additional file 3: Figure S1 shows the screening of integrated clones which were performed by serial dilution and identification of integration events by PCR. Stable transfections of *PfSWIB*-FKBP-LID integrated parasites clones *PfSWIB*-C2 and *PfSWIB*-B10 were finally obtained from the cloning and screening process in 96-well plates. Since the *PfSWIB*-C2 clone exhibited stability and reproducibility in the subsequent assays, such as PCR detection and qPCR analysis, we selected clone *PfSWIB*-C2 rather than clone *PfSWIB*-B10 for the present study.

**Fig. 2:**
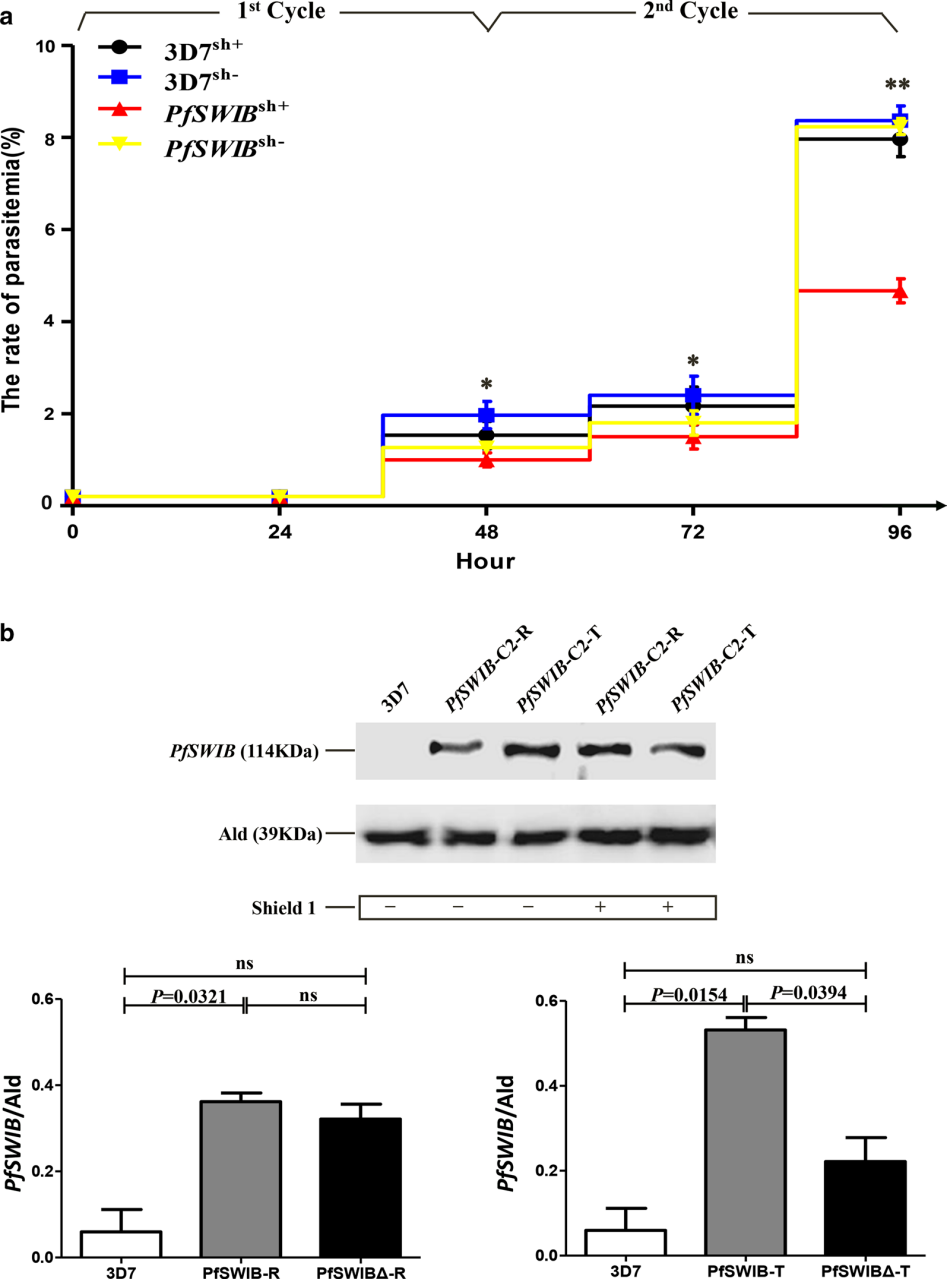
Conditional knockdown of *PfSWIB* interferes with the growth and development of parasite. **a** Growth curve analysis (GCA) during 96-h *in vitro* culturing. Data are presented as the rate of parasitemia (%) in the four parasite lines, *PfSWIB*^sh+^, *PfSWIB*^sh−^, 3D7^sh+^ and 3D7^sh−^. All parasite lines were initially synchronized into 0–6 h ring-stage parasites, with exactly the same preliminary parasitemia of 0.2%. Data were collected every 12 h up to 96 h. The survival curves are drawn using GraphPad Prism® (version 5.0). The error bars represent the mean ± SD of three independent experiments determined by counting the rate of parasitemia. *Key*: sh+, parasite lines cultured in the presence of shield1 (1:2000); sh−, lines cultured in the absence of shield1. The rate of parasitemia (%) of different lines were compared statistically by a one-way ANOVA followed by Bonferroniʼs multiple comparison *post-hoc* tests (*P < 0.05, **P < 0.01). **b** Western blot assay of *PfSWIB* fusion protein levels in three lines at the ring and trophozoite-stages within the third life-cycle of *P. falciparum*. An antibody to aldolase (Roche) was used as a positive control. Rabbit anti-HA (Abcam) was used to identify fusion *PfSWIB* proteins in different lines. *Key*: +, parasite lines cultured in the presence of shield1; −, lines cultured in the absence of shield1. Significance and different expression of *PfSWIB* fusion protein were calculated by the gray-level image analysis using ImageJ software. The error bars represent the mean ± SD of two independent western blot experiments. ‘R’ and ‘T’ denote the ring and trophozoite stage of *P. falciparum*, respectively. Statistical significance was determined with the two-tailed Student’s t-test (*P < 0.05, **P < 0.01)

Western blotting was subsequently performed to detect the expression of *PfSWIB*-HA-FKBP-LID fusion protein in the presence and absence of shield1 (1:2000, 0.5 μM). According to the previous study, *PfSWIB* is theoretically stable in the absence of the small molecule shield1 [45]. Alternatively, shield1 binds tightly to FKBP thereby substituting the degron and triggering rapid, continuous degradation of the LID domain and *PfSWIB* [45]. As shield1 may take time to exert its inhibition function, we selected the *in vitro* culturing ring and trophozoite parasite stage within their third life-cycle (> 96 h) as testing samples, according to the GCA results (Fig. 2a). The results indicated that although the *PfSWIB*-HA-FKBP-LID (114 kDa) fusion gene could be successfully expressed in both stages in the presence or absence of shield1 (Fig. 2b, Additional file 4: Figure S2a), there was still a significant difference of *PfSWIB* fusion protein expression between *PfSWIB∆* and *PfSWIB* lines in the trophozoite stage, the expression of *PfSWIB* fusion protein decreased in *PfSWIB∆* line (*t*_(2)_ = 4.890, *P* = 0.0394). In contrast, no significant difference had been found in the ring stage parasite (*t*_(2)_ = 1.006, *P* = 0.4205) (Fig. 2b, Additional file 4: Figure S2b).

### Conditional knockdown of *PfSWIB* interferes with parasite growth and development

To study the specific functions of *PfSWIB*, we subsequently analyzed the growth curve by counting parasitemia of different groups within 96 h (Fig. 2, Additional file 5: Table S3). Both *PfSWIB*-C2 and 3D7 parasite clones were initially synchronized into 0–6 h ring stage parasites and equally divided into two groups. One group contained shield1 in the culture medium, whereas the other group was lacking shield1. Thus, four groups of lines including *PfSWIB∆* (*PfSWIB* shield1 induced), *PfSWIB* (*PfSWIB*^sh−^ shield1 not induced), 3D7^sh+^ and 3D7^sh−^ were subject into the *in vitro* culture, with exactly the same preliminary parasitemia of 0.2%.

The results of GCA revealed no significant difference in parasitemia between these lines during the first 48 h life-cycle (*F*_(3,6)_ = 1.000, *P* = 0.4547). Nevertheless, the *PfSWIB∆* line showed a relatively low parasitemia during 48–84 h when compared with other lines (48 h: *F*_(3, 6)_ = 5.071, *P* = 0.0439; 72 h: *F*_(3, 6)_ = 7.775, *P* = 0.0172). Significant change was observed at 96 h (ring stage parasites within the 3rd life-cycle), the parasitemia of the *PfSWIB∆* line showed a great decline compared to other lines (*F*_(3, 6)_ = 154.3, *P* < 0.0001). Conditional knockdown of *PfSWIB* could affect parasite growth and development, suggesting that *PfSWIB* might play a key role in the proliferation of *P. falciparum*.

### Conditional knockdown of *PfSWIB* leads to a change in mutually exclusive *var* gene transcription

Based on the GCA result, we subsequently performed RNA-seq analysis in duplicate with synchronized 0–6 h ring stage parasites of three *P. falciparum* lines within their third life-cycle, to study the change of expression profiling when knocking down *PfSWIB*. The scatter plot illustrates the comparison of screening of differentially expressed genes in three parasite lines, including 3D7, *PfSWIB* and *PfSWIB*∆ (Additional file 6: Figure S3a). Among all differentially expressed genes, 112 and 450 were upregulated in the *PfSWIB*∆ line when compared with the *PfSWIB* and 3D7 line, respectively, whereas 1 and 616 genes were downregulated (fold change, FC > 1.5, *P* < 0.05, FDR < 0.05). We further performed hierarchical clustering of the individual gene expression profiles by using the R package *edgeR*. RNA-seq data were normalized for sequencing depth and gene length using log_2_-transformed FPKM values (Additional file 6: Figure S3b). According to the special characteristics of differentially expressed proteins in each line, we further screened out transcription factors such as PfApiAP2 family and PfSir2B, as well as certain histone modification enzymes, which proved to be involved in regulating on *var* gene transcription. These genes were significantly upregulated in the *PfSWIB*∆ line, suggesting the existence of their different transcription profiling when knocking down *PfSWIB*. In addition, a number of *vars* also exhibited differential expression among different lines (Additional file 2: Table S2).

**Fig. 3:**
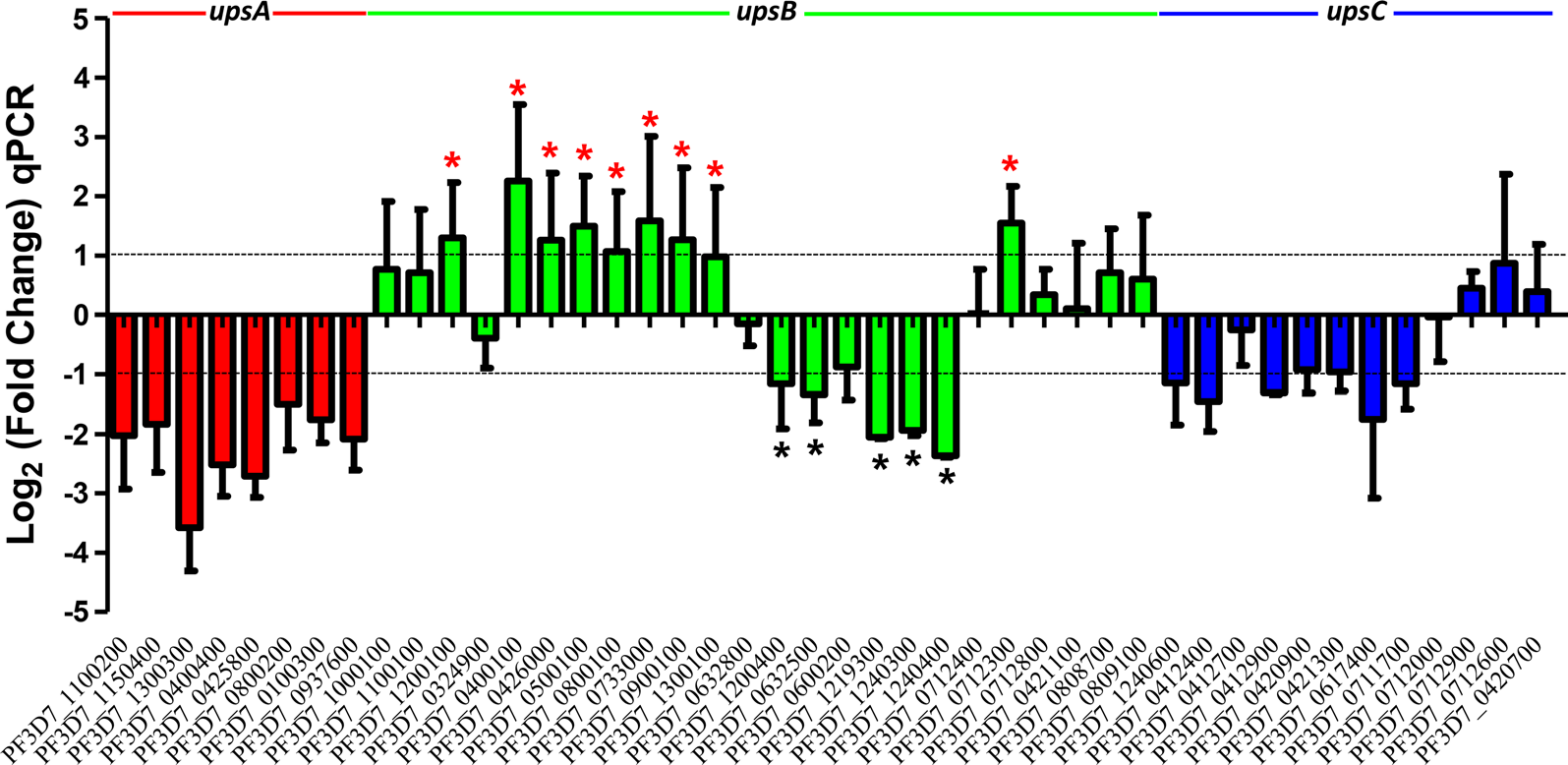
Conditional knockdown of *PfSWIB* leads to a change in mutually exclusive *var* gene transcription. qPCR analysis of the transcription level of individual *var* genes in two parasite lines, *PfSWIB* and *PfSWIB*∆. The data are presented as a log_2_ fold change of *var* gene transcription levels in the *PfSWIB*∆ line (shield1 induced) with respect to the *PfSWIB* line (shield1 not induced), using the seryl-tRNA synthetase gene (PF3D7_0717700) as an endogenous control. The error bars represent the mean ± SD of three independent experiments. The red, green and blue bars in the histogram represent *upsA*-, *upsB*- and *upsC*-type *vars*. Red asterisks: 9 upregulated *upsB vars* (log_2_ fold change ≥ 1.0, *P* < 0.05), black asterisks: 5 downregulated *upsB vars* (log_2_ fold change ≤ − 1.0, *P* < 0.05)

To further study the transcription patterns of *upsA-*, *upsB*- and *upsC*-subtype *var* genes in different parasite lines, we first selected 44 differentially expressed *vars* among different lines based on the RNA-seq results (fold change, FC > 1.5, *P* < 0.05, FDR < 0.05), and tested the expression patterns of these preferred *vars* in each comparison group at the ring stage (0–10 h after re-invasion in 3rd life-cycle, > 96 h) by real-time PCR (Additional file 2: Table S2). *upsB* or *upsA* corresponds to subtelomeric and *upsC* corresponds to chromosome internal *var* gene members [55, 56]. Figure 3 shows the fold change of *var* gene expression profiling between shield1 pre- and post-induced lines *PfSWIB*∆ *vs PfSWIB*. Most *upsA*- and *upsC-* type *vars* were significantly downregulated in the *PfSWIB*∆ line, especially the *upsA vars*, when compared to the *PfSWIB* line (Fig. 3, Additional file 7: Figure S4). However, the expression pattern of partial *upsB vars* presented a distinct change in the *PfSWIB*∆ line. A group of 9 *upsB vars* exhibited a high level of expression (Fig. 3, red asterisk) (fold change ≥ 2.0, *P* < 0.05), while 5 *upsB vars* present an extremely low expression level (Fig. 3, black asterisk) (fold change ≤ − 2.0, *P* < 0.05), when compared the *PfSWIB*∆ line to other lines.

**Fig. 4:**
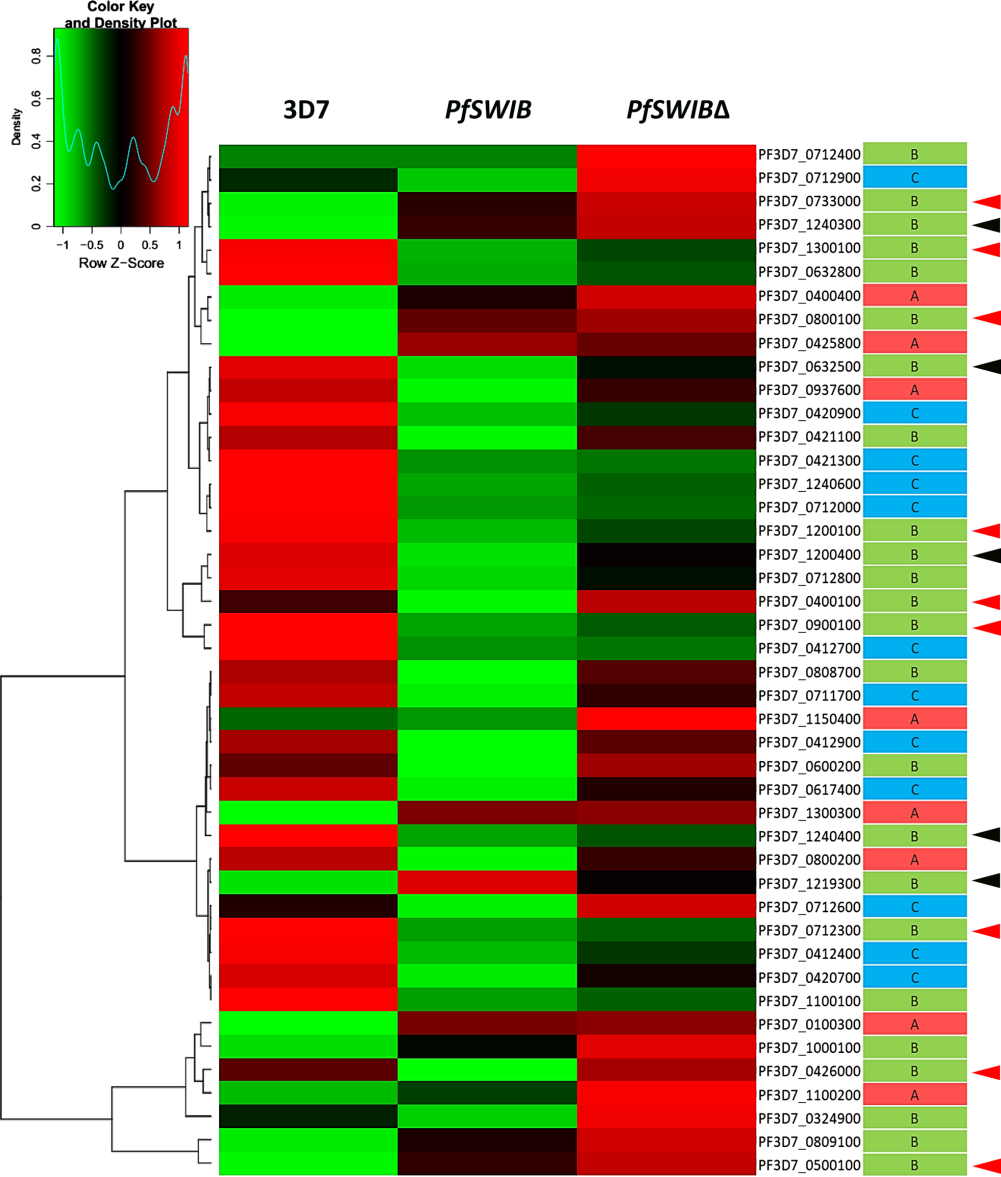
Conditional knockdown of *PfSWIB* leads to a change in expression profiles of 44 *vars* in different lines. Hierarchical clustering heatmap of log_2_ transformed fragments per kilobase of transcript per million (FPKM) gene expression values for 44 *var* genes after re-invasion in the 3rd life-cycle of *P. falciparum*. The color scale ranges from green to red, showing a range from minimum (≤ − 0.4) to maximum (≥ − 0.4) log_2_ FPKM gene expression values for each *var* variant. Row labels with red and black arrows denote 9 upregulated and 5 downregulated *upsB vars* detected by qPCR analysis, respectively. Different *var* gene subtypes are shown in differently colored boxes

We subsequently re-analyzed the RNA-seq results of *var*s in these lines by using the R package *edgeR*, to identify similarities and differences in expression patterns for different *var* variants. The heatmap representing the hierarchical clustering of the *var* genes is shown in Fig. 4 and Additional file 8: Figure S5. Figure 4 indicates two major clusters of 44 selected *vars* in relation to the expression pattern: one major cluster consisting of 37 *var* variants covered most of *upsA*, *upsB* and all *upsC vars*, while the other cluster covered only 5 *upsB* and 2 *upsA vars*. In addition, there were 24 and 20 *vars* variants showed an expression maximum in the 3D7 line and *PfSWIB*∆ line, respectively (red box). Additional linear regression analysis of the correlation between qPCR and RNA-seq data was performed to investigate the transcriptome data accuracy on 44 *var* genes (Additional file 9: Figure S6, Additional file 10: Table S4). In the *PfSWIB vs PfSWIB∆* comparison, the qPCR data showed a significant linear correlation with the RNA-seq data (*r*_(44)_ = 0.6281, *P* < 0.0001). The RNA-seq results of 9 upregulated and 5 downregulated *upsB vars* (described above by qPCR analysis in the *PfSWIB*∆ line), were also re-analyzed by computing the log_2_ fold change of FPKM values in different lines. Of these, 5 out of 9 upregulated *upsB vars* (except for PF3D7_1300100, PF3D7_1200100, PF3D7_0900100 and PF3D7_0712300) (Fig. 4, red arrow), and 4 out of 5 downregulated (except for PF3D7_1240300) (Fig. 4, black arrow) showed consistency with the qPCR results. Additional file 8: Figure S5 shows the expression pattern and hierarchical clustering of all the 60 *vars* in the three lines.

### Conditional knockdown of *PfSWIB* leads to a change in mutually exclusive *var* gene transcription in a single 48 hour life-cycle

According to previous studies, *var* genes were highly expressed in the ring stage, while drastically downregulated in the trophozoite stage [6, 10, 11]. To confirm this, we initially collected RNA samples from the ring and trophozoite stages of the 3D7 clone within the third life-cycle of *P. falciparum*, and analyzed the *var* gene expression pattern by observing the log_2_ fold change of trophozoite to ring in the absence and presence of shield1 (1:2000, 0.5 μM). Approximately all of the *var* genes, were downregulated at the mature stage of 3D7^sh+^ and 3D7^sh−^ parasite lines, except for PF3D7-0632500 (*upsA*), and PF3D7_1219300 and PF3D7_0712900 (*upsB*), suggesting that shield1 may have no influence on the expression of most *var* genes in a single 48 hour-life cycle of *P. falciparum* (Additional file 11: Figure S7).

**Fig. 5:**
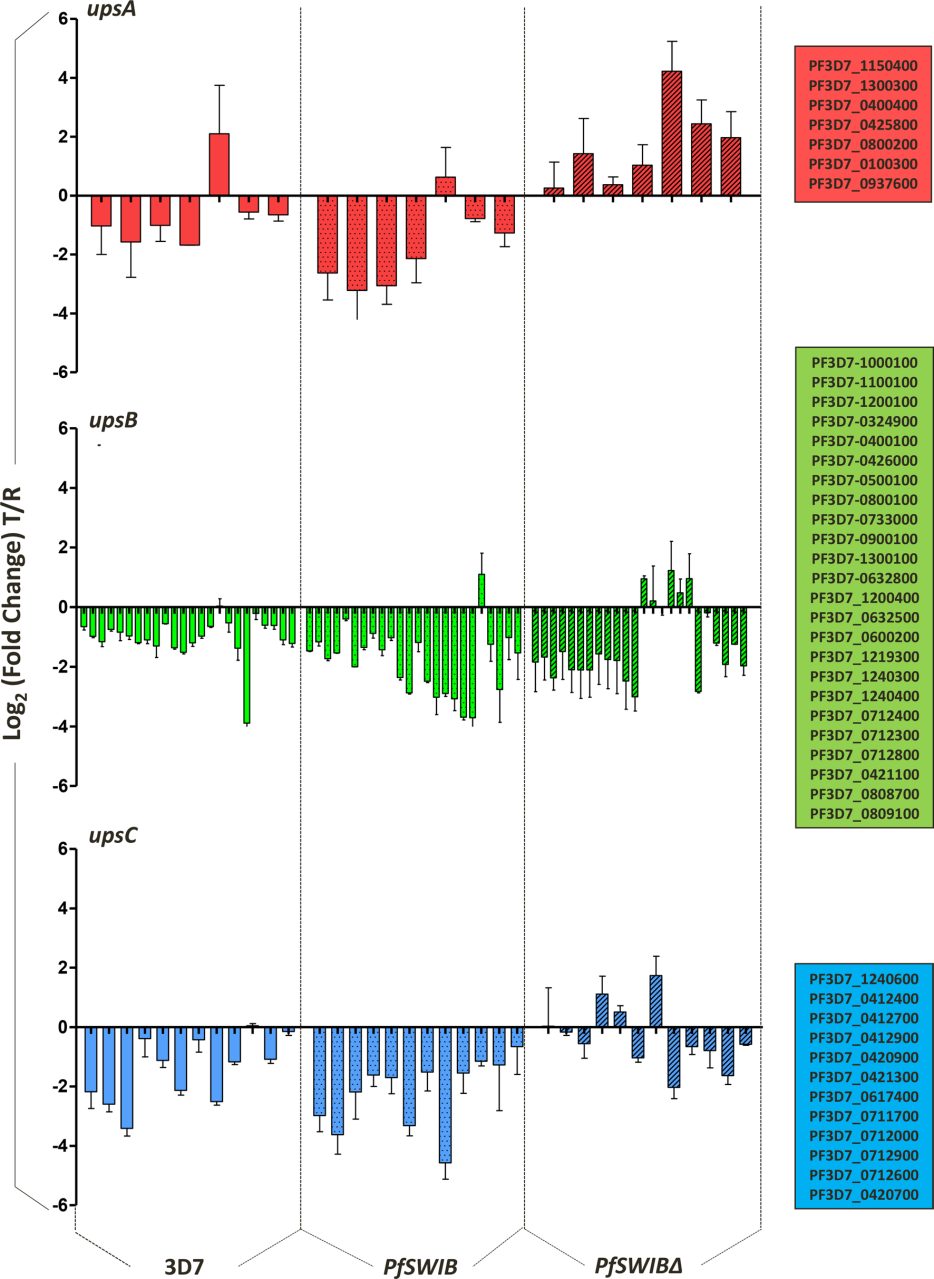
Conditional knockdown of *PfSWIB* leads to a change in mutually exclusive *var* gene transcription in a single 48-h life-cycle. qPCR analysis of the transcription level of individual *var* genes in three lines during a single 48-h life-cycle of *P. falciparum.* The data are presented as log_2_ fold change of trophozoite to ring within the third life-cycle, using the seryl-tRNA synthetase gene (PF3D7_0717700) as an endogenous control. The error bars represent the mean ± SD of three independent experiments. The red, green and blue bars in the histogram represent *upsA*-, *upsB*- and *upsC*- type *vars*, respectively

We subsequently analyzed the log_2_ ratio of T/R in *invitro* cultured *PfSWIB∆* and *PfSWIB* lines within their third life-cycle after collecting RNA samples from their synchronized ring and trophozoite parasites. Among these parasite lines, *PfSWIB* exhibited a quite similar log_2_ ratio of T/R to 3D7, which was characterized by the downregulation of most *var* genes at the mature stage (Fig. 5). In contrast, approximately all of the *upsA vars*, as well as partial *upsB* (PF3D7_1200400, PF3D7_0632500, PF3D7_1219300, PF3D7_1240300 and PF3D7_1240400) and *upsC* (PF3D7_0412900, PF3D7_0420900 and PF3D7_0617400) *vars* were activated at the mature stage of *P. falciparum* in the *PfSWIB∆* line (Fig. 5).

## Discussion

The interplay between altered histone modification, chromatin modifying machinery, and general or sequence-specific transcription factors results in *var* gene regulation by modulating chromatin structure [57]. In this process, ATP-dependent chromatin remodeling complexes (CRCs) play a key role in chromatin remodeling through removal and movements of nucleosomes and deposition of alternative histones [13]. There are at least 11 proteins containing putative SWI2/SNF2 ATPase catalytic domains in *P. falciparum* [58], seven of which are predicted to be chromatin-modifying proteins, including ISWI, Swr1, CHD1, SNF2L, MAL8P1.65, PFB0730w, PFF0225w and PF13_0308 [18]. However, it remains unclear how these PfCRCs regulate *var* gene expression by remodeling certain chromatin and therefore resulting in a high level of antigenic variation, immunity evasion and pathopoiesis.

The SWI/SNF complex, originally revealed in yeasts, allows for transcriptional activation by remodeling chromatin [59]. It has also been demonstrated previously that mammalian SWI/SNF homologs complexes, also known as BRG1-associated factors (BAFs), can be found in multiple forms which consist of 9–12 proteins. Among these BAFs, BAF60b exists in an alternative form of the SWI/SNF complex (Complex B), which is generally known as SWI-B or SWIB [34]. In humans, the BAF60a protein of the SWI/SNF complex is responsible for p53 binding and regulates its activities *via* an N-terminal region of the SWIB/MDM2 domain [35], as well as facilitating cell cycle halting and tune the balance between repair and apoptosis induction [35–37]. SWIB/MDM2 domains have in fact been shown to participate in protein-protein [60] and chromatin-related interactions [61], but their functional roles in other species are poorly understood. Similarly, the SWI/SNF complex is composed of constant units and includes the Swp73p/SNF12 protein comprising a SWIB/MDM2 domain, proposed to facilitate specificity and/or functionality to some extent [38, 62]. According to recent studies, the *P. falciparum* genome encodes two putative SWIB/MDM2 domain-containing proteins: *PfMDM2* (PF3D70518200, SWIB/MDM2 domain-containing protein) and *PfSWIB* (PF3D70611400, SWI/SNF-related matrix-associated actin-dependent regulator of chromatin) [38], and *PfSWIB* was not only crucial for the survival and growth of the parasite, but additionally had profound effects on the parasite by interacting with other unknown proteins [38–40]. Bioinformatics analysis demonstrated that *PfSWIB* is characterized by its SWIB domain (BAF60b domain, PlasmoDB, http://plasmodb.org/). Bonger et al. [45] have recently developed a new technique by which the degradation of a specific protein is definitely induced by way of a small molecule, shield1. Hence, an in-depth functional analysis of *PfSWIB* based on the conditional knock-down assay was implemented in the present study. By using a transgenic assay, drug screening and subsequent screening of the clones, we obtained two integrated parasite clones *PfSWIB*-C2 and B10, with a stable transfection of the modified locus *PfSWIB*-FKBP-LID into the *Plasmodium* parasites (Fig. 1, Additional file 3: Figure S1). Western blot analysis confirmed that the fusion expression protein was successfully expressed in the transfected parasite lines, and there was a significant decrease of *PfSWIB* fusion protein expression in the *PfSWIB∆* line (*t*_(2)_ = 4.890, *P* = 0.0394), at its trophozoite stage within third life-cycle of *P. falciparum*. However, it should be noted that no significant change was detected in the protein expression level of *PfSWIB* fusion system during the ring stage, either in the presence or absence of drug pressure (*t*_(2)_ = 1.006, *P* = 0.4205) (Fig. 2b, Additional file 4: Figure S2). This could be due to at least two reasons. First, it takes time for shield1 to work at full capacity through binding to LID, therefore, a portion or slight loss of fused *PfSWIB* protein could not be identified. Secondly, this protein might be expressed predominantly at the mature stage of *P. falciparum*.

It is worth noting that *PfSWIB* may lead to stage-specific PCD, in the same way as BAF60a of the mammalian SWI/SNF complex induces p53-directed apoptosis, by which transient nuclear localization results in removal of these parasites, as previously described [35]. Vieira et al. [38] further demonstrated that *PfSWIB* could have a stage-specific, pro-survival function and takes part in the heat stress response of the *Plasmodium* parasite. They discovered that *PfSWIB* and *PfARK3* participate in preventing p53-mediated apoptosis within the nucleus in reaction to heat stress [38]. Alternatively, human Aurora kinase B and its yeast homologue play key roles during gene transcription through histone H3 phosphorylation [39, 40]. Likewise, *PfSWIB* could also have an effect on parasite survival through transcriptional regulation by guiding the *PfARK3*’s phosphorylation activities [39, 40]. In our study, growth curve analysis of *P. falciparum* revealed that a relatively low parasitemia was observed in the *PfSWIB* knockdown line (*PfSWIB∆*) when compared to other parasite lines during their second life-cycle. Significant change was observed after 96 hours, when the parasites developed into the third life-cycle. The parasitemia of *PfSWIB∆* showed a sharp decline (Fig. 2a), suggesting that *PfSWIB* might play a key role in the proliferation and survival of *P. falciparum*.

On the other hand, Jiang et al. [27] proved that knocking out the *P. falciparum* variant-silencing SET gene, which encodes an ortholog of *Drosophila melanogaster* ASH1 and controls histone H3 lysine 36 trimethylation (H3K36me3) on *var* genes, leads to expression of all *vars*. In another study focused on a novel chromatin-associated exoribonuclease, termed PfRNase II, Zhang et al. [63] revealed that this PfRNase II controls the silencing of *upsA var* genes, by marking their transcription start site and intron promoter regions leading to short-lived cryptic RNA. Depletion of PfRNase II specifically removes the silencing of *upsA vars*. Similarly, in the present study, knocking down of *PfSWIB* results in the silencing of *upsA*, *upsC* and partial *upsB var* genes, as well as removes the silencing of partial *upsB vars* (Fig. 3, Additional file 7: Figure S4). Therefore, *PfSWIB* might be one of the trans factors activating *var* genes, especially the *upsA vars*, and silencing partial *upsB vars*. In addition, a few *var* genes exhibited inconsistent RNA-seq results with qPCR, especially the *upsA vars*. They are more likely to be conditionally activated in the *PfSWIB* knockdown line, according to the RNA-seq analysis (Fig. 4, Additional file 8: Figure S5). The minor inconsistency between the RNA-seq and the qPCR results for partial *vars* could be due to false positives/negatives resulting from either of these two methods, or experimental variation caused by different parasite lines or other unidentified factors. Nevertheless, by comparing the log_2_ fold change data from qPCR and RNA-seq on 44 *var* genes, we confirmed that the linear regression and linear correlation analysis showed a significant linear correlation between the two methods (*r*_(44)_ =v0.6281, *P* < 0.0001) (Additional file 9: Figure S6).

Furthermore, previous studies have demonstrated that *var* genes were highly expressed in the ring stage of *P. falciparum*, while drastically downregulated in the trophozoite stage. The *var* transcriptional levels in trophozoites are usually 10–100 times less than those in rings [6, 7, 10, 11]. A single *P. falciparum* parasite simultaneously transcribes multiple *var* genes but selects only one PfEMP-1 to attain the surface of the host cell by way of a developmentally regulated process [6, 7]. In order to test whether the transcription pattern had been disrupted when knocking down *PfSWIB*, we subsequently compared the transcription level of selected *var* genes between ring and trophozoite stage parasites, by testing the log_2_ fold change of T/R in each group during a single life-cycle of *P. falciparum* (Fig. 5, Additional file 11: Figure S7). Strikingly, we discovered that most of the *upsA*, *upsB* and *upsC vars* were downregulated in *PfSWIB* and 3D7 lines, while virtually all the *upsA vars* and partial *upsB/upsC vars* were significantly upregulated at the trophozoite-stage of the *PfSWIB∆* line within the third life-cycle of *P. falciparum* (Fig. 5), suggesting that *PfSWIB* might be involved in temporal regulation of *var* genes, particularly group A *vars*. The dysfunction of *PfSWIB* could disrupt the interactions between *PfSWIB* and other transcription factors which are essential for gene silencing or inhibit *PfSWIB* activity through unknown mechanisms.

As we mentioned previously, *P. falciparum* varies its surface protein expression, particularly the *var* gene family-encoded clonally variant surface protein expression, to evade host immune responses [6]. In the process of monoallelic expression, specific silent *var* genes are usually retained at unique perinuclear sites and relocated to transcriptionally active zones [21, 28]. Nuclear actin has been discovered complexed with *PfSET10*, which has been suggested to play a role in the movement of the locus during activation and heterochromatic silencing [26, 29]. Actin and actin-related proteins (Arps) are common components of chromatin remodeling complexes and are involved in nuclear positioning. According to biological characteristics, *PfSWIB* may be involved in the process of relocating episomes, as an actin-dependent regulator of chromatin. Further studies regarding the possible role of *PfSWIB* and actin interaction would be worthwhile.

## Conclusions

In the present study, we revealed that actin-related chromatin remodeling factor *PfSWIB* is involved in the process of clonal variation in *var* gene expression, and is crucial for the survival and growth of the *Plasmodium* parasite. Depletion of *PfSWIB* not only silences *upsA*, *upsC* and partial *upsB var* genes and removes the silencing of partial *upsB var* genes, but also leads to the aberrant expression of *upsA* and partial *upsB/upsC var* genes at the mature stage of *P. falciparum*, during a single 48-hour life-cycle. These findings could provide a better understanding of the regulatory function of PfCRCs in regulating the clonal variation of the *var* gene family contributing to the pathogenesis in malaria parasites.

## Supplementary information

**Additional file 1: Table S1.** Specific primers used for detecting integration events.

**Additional file 2: Table S2.** Primer sets used in qPCR assays specifically to amplify 44 *var* genes.

**Additional file 3: Figure S1.** Identification of integration events and clone screening of integrated clones. **a** Clone screening was performed by a serial dilution of parasites in 96-well plates. **b** Identification of integration events by PCR.

**Additional file 4: Figure S2.** Western blot of *PfSWIB* in different parasite lines. **a** Western blot was performed within the third life-cycle. **b** Gray-level image analysis was performed using ImageJ software. *Abbreviations*: R, ring; T: trophozoite; Ald: aldolase.

**Additional file 5: Table S3.** Growth curve analysis during 96 h *in vitro* culturing.

**Additional file 6: Figure S3.** RNA-seq analysis of expression profile in different parasite lines. **a** Comparison of transcriptomes after re-invasion in 3rd life-cycle. **b** Hierarchical clustering heatmap for all the differentially expressed genes after re-invasion in 3rd life-cycle.

**Additional file 7: Figure S4.** Conditional knockdown of *PfSWIB* leads to a change in mutually exclusive *var* gene transcription. Data are presented as fold change of *var* transcription level in the *PfSWIB****∆*** line with respect to the *PfSWIB* line.

**Additional file 8: Figure S5.** Conditional knockdown of *PfSWIB* leads to a change in expression profile of 60 *vars* in different lines.

**Additional file 9: Figure S6.** Linear regression analysis between qPCR and RNA-seq data. Red dots denote the log_2_ (fold change) of 44 *vars* in the *PfSWIB vs PfSWIB∆* comparison.

**Additional file 10: Table S4.** Log_2_ (fold change) qPCR and RNA-seq data of 44 *vars* in the *PfSWIB vs PfSWIB*∆ comparison.

**Additional file 11: Figure S7.** Detection of *Var* gene expression pattern during a single 48-hour life-cycle in *P. falciparum* clone 3D7. *Abbreviations*: R, ring; T, trophozoite; *Key*: +, shield1 induced; -, shield1 not induced.

## Data Availability

Data supporting the conclusions of this article are included within the article and its additional files. The datasets generated and/or analysed during the present study are available in the GEO repository: https://www.ncbi.nlm.nih.gov/geo/query/acc.cgi?acc=GSE141155 with accession number GSE141155, and https://www.ncbi.nlm.nih.gov/geo/query/acc.cgi?acc=GSE141404, with accession number GSE141404. The above two subseries are linked to the SuperSeries GSE141959 (https://www.ncbi.nlm.nih.gov/geo/query/acc.cgi?acc=GSE141959). The raw datasets used and/or analysed during this study are available from the corresponding author upon reasonable request.

## Abbreviations

PCD: programmed cell death
BSD: blasticidin S deaminase
GCA: growth curve analysis
CM: cerebral malaria
PAM: pregnancy-associated malaria
PfEMP1: *Plasmodium falciparum* erythrocyte membrane protein 1
H3K9ac: histone 3 lysine 9 acetylation
H3K4me3: histone 3 lysine 4 trimethylation
TSS: transcriptional start site
H3K9me3: histone 3 lysine 9 trimethylation
H3K36me3: histone 3 lysine 36 trimethylation
HATs: histone acetyltransferases
HDAC: histone deacetylases
HKMTs: histone lysine methyltransferases
HAS: helicase-SANT
CRCs: chromatin remodeling complexes
HA: hemagglutinin
FKBP: FK506- and rapamycin-binding protein
LID: ligand-induced degradation domain
ARPs: actin related proteins
