# Supplementary material for: PfSWIB, a potential chromatin regulator for var gene regulation and parasite development in Plasmodium falciparum
Source: Parasit Vectors. 2020 Feb 4;13:48. doi: 10.1186/s13071-020-3918-5 (PMC7001229; doi:10.1186/s13071-020-3918-5)
Supplement: Supplementary file 1 — Additional file 1: Table S1. Specific primers used for detecting integration events. [file 13071_2020_3918_MOESM1_ESM.docx]

**Additional file 1: Table S1. Specific primers used for detecting integration events.**

| **Primer** | **Sequence** |
| --- | --- |
| *PfSWIB*-Forward | GCGCGGGGCCCGTTATTGGAACTTTTAAGAATTAAT |
| *PfSWIB*-Reverse | GGCGCGCCAAAAATTATTATTATTATTATTATTATTATTATT |
| P2^*^ | AAAGGTAATAGCATTTTTGTGTCC |
| P4^*^ | GAAAATTCTGAACAATCGGAGAG |
| P5^*^ | TGTAGACCCCATTGTGAGTAC |
| P2’^**^ | GAAATGAAAAATGAAATTTGTACAC |
| P4’^**^ | GTATATTAGAGGTAGACGAATATG |
| P5’^**^ | CCACGTCGTCTAAGTAAAACTAC |
| *bsd*-Forward^a^ | ATGGTGATAAATGCATGCCA |
| *bsd*-Reverse^a^ | GCCCTCCCACACATAACCAG |
| *actin*-Forward^b^ | ATGGGAGAAGAAGATGTTCAAG |
| *actin*-Reverse^b^ | GAAACATTTTCTGTGGACAATAC |

*. Primer specific to 3’UTR of endogenous *PfSWIB* gene, the upstream sequence of *PfSWIB* C-terminus and downstream plasmid sequence of HA-FKBP-LID fusion expression system, respectively.

**. The alternative P2, P4 and P5 specific primers for qPCR analysis.

a. Drug selectable marker gene blasticidin deaminase.

b. Endogenous control: actin1 (GenBank: PF3D7_1246200).
