# Supplementary material for: PfSWIB, a potential chromatin regulator for var gene regulation and parasite development in Plasmodium falciparum
Source: Parasit Vectors. 2020 Feb 4;13:48. doi: 10.1186/s13071-020-3918-5 (PMC7001229; doi:10.1186/s13071-020-3918-5)
Supplement: Supplementary file 2 — Additional file 2: Table S2. Primer sets used in qPCR assays specifically to amplify 44 var genes. [file 13071_2020_3918_MOESM2_ESM.docx]

**Additional file 2: Table S2. Primer sets used in qPCR assays specifically to amplify 44 *var* genes*.**

| **Gene ID** | **Previous ID** | **Forward primer** | **Reverse primer** |
| --- | --- | --- | --- |
| PF3D7_1100200^a^ | PF11_0008 | GACGGCTACCACAGAGACAA | CGTCATCATCGTCTTCGTTT |
| PF3D7_1150400^a^ | PF11_0521 | TGCTGAAGACCAAATTGAGC | TTGTTGTGGTGGTTGTTGTG |
| PF3D7_1300300^a^ | PF13_0003 | CACAGGTATGGGAAGCAATG | CCATACAGCCGTGACTGTTC |
| PF3D7_0400400^a^ | PFD0020c | ATATGGGAAGGGATGCTCTG | TGAACCATCGAAGGAATTGA |
| PF3D7_0425800^a^ | PFD1235w | AAACACGTTGAATGGCGATA | GACGCCGAGGAGGTAAATAG |
| PF3D7_0800200^a^ | PF08_0141 | GGTGTCAAGGCAGCTAATGA | TATGTCCTGCGCTATTTTGC |
| PF3D7_0100300^a^ | PFA0015c | TCATTATGGGAAGCACGATT | TGATTTCTACCATCGCAAGG |
| PF3D7_0937600 ^a^ | PFI1820w | TGACCAAGACGAAGTATGGAA | TTGATCTCTGTTCGCTGTCC |
| PF3D7_1000100^b^ | PF10_0001 | GACGAGGAGTCGGAAAAGAC | TGGACAGGCTTGTTTGAGAG |
| PF3D7_1100100^b^ | PF11_0007 | GAGGCTTATGGGAAACCAGA | AGGCAGTCTTTGGCATCTTT |
| PF3D7_1200100^b^ | PFL0005w | CGGAGGAGGAAAAACAAGAG | TGCCGTATTTGAGACCACAT |
| PF3D7_0324900^b^ | PFC1120c | CAATCTGCGGCAATAGAGAC | CCACTGTTGAGGGGTTTTCT |
| PF3D7_0400100^b^ | PFD0005w | GACGACGATGAAGACGAAGA | AGATCTCCGCATTTCCAATC |
| PF3D7_0426000^b^ | PFD1245c | TGACGACTCCTCAGACGAAG | CTCCACTGACGGATCTGTTG |
| PF3D7_0500100^b^ | PFE0005w | GAAGCTGGTGGTACTGACGA | TATTTTCCCACCAGGAGGAG |
| PF3D7_0800100^b^ | PF08_0142 | GTCGTGGAAAAACGAAAGGT | TATCTATCCAGGGCCCAAAG |
| PF3D7_0733000^b^ | PF07_0139 | TGACGACGATAAATGGGAAA | TTCTTTTGGAGCAGGGAGTT |
| PF3D7_0900100^b^ | PFI0005w | TGCAAACCACCAGAAGAAAG | GTTCTCCGTGTTGTCCTCCT |
| PF3D7_1300100^b^ | PF13_0001 | ACAAAGGAACGTCCATCTCC | GCCAATACTCCACATGATCG |
| PF3D7_0632800^b^ | PFF1595c | GACAAATACGGCGACTACGA | TGTTTCACCCCATTCTTCAA |
| PF3D7_1200400^ba^ | PFL0020w | TCGATTATGTGCCGCAGTAT | TTCCCGTACAATCGTATCCA |
| PF3D7_0632500^ba^ | PFF1580c | ATGTGTGCGAGAAGGTGAAG | TGCCTTCTAGGTGGCATACA |
| PF3D7_0600200^ba^ | PFF0010w | TGGAAAGAACATGGACCTGA | TTCCTCGAGGGAAGAATCAC |
| PF3D7_1219300^bc^ | PFL0935c | GACGCCTGCACTCTCAAATA | TTGGAGAGCACCACCATTTA |
| PF3D7_1240300^bc^ | PFL1950w | AGCAAAATCCGAAGCAGAAT | CCCACAGATCTTTTCCTCGT |
| PF3D7_1240400^bc^ | PFL1955w | AAAGCCACTAGCGAGGGTAA | TGTTTTTGCCCACTCCTGTA |
| PF3D7_0712400^bc^ | PF07_0050 | GCGACGCTCAAAAACATTTA | TCATCCAACGCAATCTTTGT |
| PF3D7_0712300^bc^ | MAL7P1.50 | ACCAAATGGTGACTTGCTCA | TTTTCATCGACGGATGATGT |
| PF3D7_0712800^bc^ | MAL7P1.55 | ACGTGGTGGAGACGTAAACA | CCTTTGTTGTTGCCACTTTG |
| PF3D7_0421100^bc^ | PFD1005c | ACCAAGTGGTGACAAAGCAG | GGGTGGCACACAAACACTAC |
| PF3D7_0808700^bc^ | PF08_0106 | TTTGTCCGGAAGACGATACA | ATCTGGGGCAGAATTACCAC |
| PF3D7_0809100^bc^ | PF08_0103 | TGCAAGGGTGCTAATGGTAA | CCTGCATTTTGACATTCGTC |
| PF3D7_1240600^c^ | PFL1960w | CATCCATTACGCAGGATACG | AAATAGGGTGGGCGTAACAC |
| PF3D7_0412400^c^ | PFD0615c | ACCGCCCCATCTAGTGATAG | CACTTGGTGATGTGGTGTCA |
| PF3D7_0412700^c^ | PFD0625c | TAAAAGACGCCAACAGATGC | TCATCGTCTTCGTCTTCGTC |
| PF3D7_0412900^c^ | PFD0630c | ACTTTCTGGTGGGGAATCAG | TTCACCGCCACTTACTTCAG |
| PF3D7_0420900^c^ | PFD1000c | AGAGGGTTATGGGAATGCAG | GCATTCTTTGGCAATTCCTT |
| PF3D7_0421300^c^ | PFD1015c | TGCAACGAAACATTAGCACA | AGCAGGGGATGATGCTTTAC |
| PF3D7_0617400^c^ | PFF0845c | ATTTGTCGCACATGAAGGAA | AACTTCGTGCCAATGCTGTA |
| PF3D7_0711700^c^ | PF07_0048 | CAATTTTTCCGACGCTTGTA | CACATATAGCGCCGTCCTTA |
| PF3D7_0712000^c^ | PF07_0049 | GTTGAGTCTGCGGCAATAGA | CTGGGGTTTGTTCAACACTG |
| PF3D7_0712900^c^ | MAL7P1.56 | CACACATGTCCACCACAAGA | ACCCTTCTGTGGTGTCTTCC |
| PF3D7_0712600^c^ | PF07_0051 | CGTGGTAGTGAAGCACCATC | CCCACCTTCTTGTGGTTTCT |
| PF3D7_0420700^c^ | PFD0995c | TCACAACCTGACCCCCTACT | TCTTCGTCGTTGTCATCCTC |
| PF3D7_717700^d^ | PF07_0073 | AAGTAGCAGGTCATCGTGGTT | TTCGGCACATTCTTCCATAA |

**var* primer pairs for detecting individual *var* gene expression were designed based on published literature [48].

a. *upsA*-subtype *var* genes

b/ba/bc. *upsB*-subtype *var* genes

c. *upsC*-subtype *var* genes

d. endogenous control: seryl-tRNA synthetase gene (GenBank: PF3D7_0717700)
