# Supplementary material for: PfSWIB, a potential chromatin regulator for var gene regulation and parasite development in Plasmodium falciparum
Source: Parasit Vectors. 2020 Feb 4;13:48. doi: 10.1186/s13071-020-3918-5 (PMC7001229; doi:10.1186/s13071-020-3918-5)
Supplement: Supplementary file 3 — Additional file 3: Figure S1. Identification of integration events and clone screening of integrated clones. a Clone screening was performed by a serial dilution of parasites in 96-well plates. b Identification of integration events by PCR. [file 13071_2020_3918_MOESM3_ESM.docx]

**
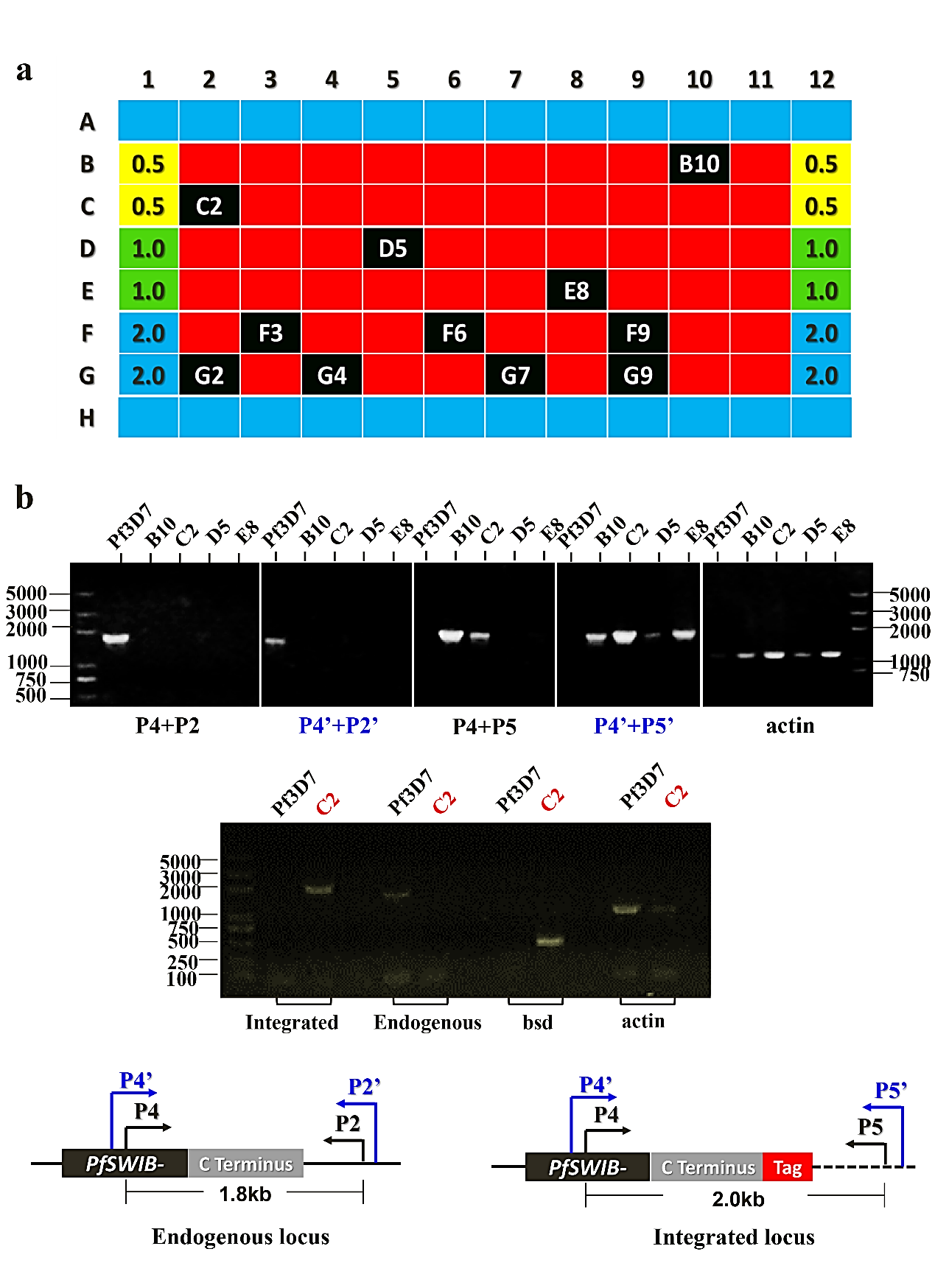
**

**Additional file 3: Figure S1.** Identification of integration events and clone screening of integrated clones. **a** Clone screening of integrated clones. Clone screening was performed by a serial dilution of parasites in 96-well plates. B10, C2, D5, E8, F3, F6, F9, G2, G4, G7 and G9 represent different clones at a level of 0.5, 1 and 2 parasite per well, detected by microscopy. **b** Identification of integration events by PCR. The integration events were detected on screened clones at a level of 0.5 and 1 parasite per well after extended the culture to 24-well plates. The description of primers P2, P4 and P5 can be found in Additional file 1: Table S1. P4 + P2 are used for detecting the endogenous *PfSWIB* locus, while P4 + P5 are used for detecting the integrated *PfSWIB* locus. P2’, P4’ and P5’ are alternative primers in respect to P2, P4 and P5, respectively.
