# Supplementary material for: PfSWIB, a potential chromatin regulator for var gene regulation and parasite development in Plasmodium falciparum
Source: Parasit Vectors. 2020 Feb 4;13:48. doi: 10.1186/s13071-020-3918-5 (PMC7001229; doi:10.1186/s13071-020-3918-5)
Supplement: Supplementary file 4 — Additional file 4: Figure S2. Western blot of PfSWIB in different parasite lines. a Western blot was performed within the third life-cycle. b Gray-level image analysis was performed using ImageJ software. Abbreviations: R, ring; T: trophozoite; Ald: aldolase. [file 13071_2020_3918_MOESM4_ESM.docx]

**
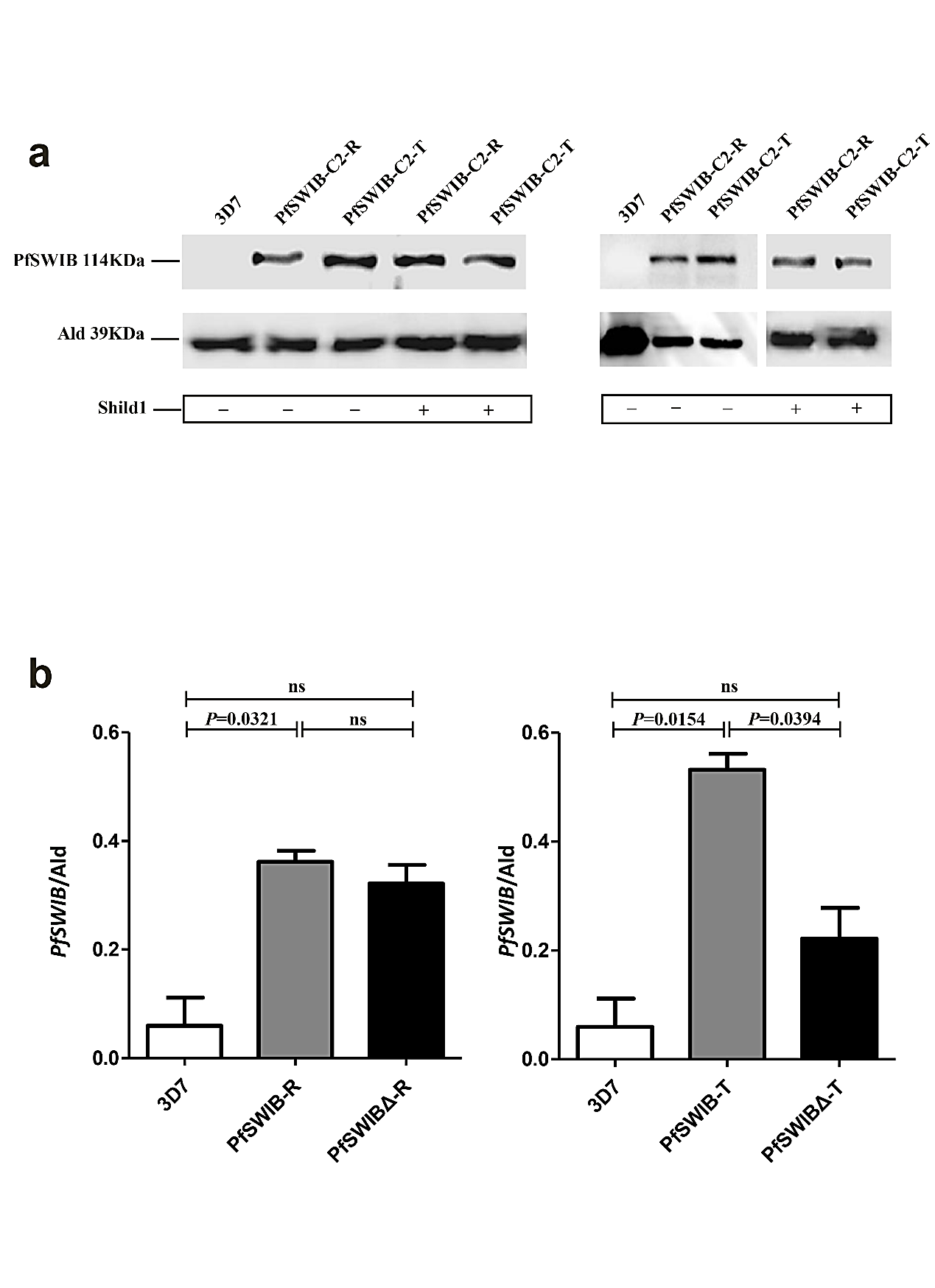
**

**Additional file 4: Figure S2.** Western blot of *PfSWIB* in different parasite lines. **a** Western blot assay of *PfSWIB* fusion protein levels in three parasite lines at the ring and trophozoite-stages within the third life-cycle of *P. falciparum*. An antibody to aldolase (Roche) was used as a positive control. Rabbit anti-HA (Abcam) was used to identify fusion *PfSWIB* proteins in different lines. *Key*: +, shield1 induced; -, shield1 not induced. The western blot was repeated twice. **b** Significance and different expression of *PfSWIB-HA-FKBP-LID* (114 kDa) fusion protein were calculated by gray-level image analysis using ImageJ software. The error bars represent the mean ± SD of two independent western blot experiments. Statistical significance was determined using a two-tailed Student’s t-test (**P*< 0.05, ***P*< 0.01). *Abbreviations*: R, ring; T: trophozoite; Ald: aldolase.
