# Supplementary material for: PfSWIB, a potential chromatin regulator for var gene regulation and parasite development in Plasmodium falciparum
Source: Parasit Vectors. 2020 Feb 4;13:48. doi: 10.1186/s13071-020-3918-5 (PMC7001229; doi:10.1186/s13071-020-3918-5)
Supplement: Supplementary file 5 — Additional file 5: Table S3. Growth curve analysis during 96 h in vitro culturing. [file 13071_2020_3918_MOESM5_ESM.docx]

**Additional file 5: Tables S3.** **Growth Curve Analysis during 96 hours in-vitro culturing.**

| **GCA Repeat1** | **0h** | **24h** | **48h** | **72h** | **96h** |
| --- | --- | --- | --- | --- | --- |
| **3D7^sh+^** | 0.20 | 0.20 | 1.40 | 2.30 | 7.80 |
| **3D7^sh-^** | 0.20 | 0.20 | 2.10 | 2.60 | 8.40 |
| ***PfSWIB*^sh+^** | 0.20 | 0.20 | 1.10 | 1.60 | 4.70 |
| ***PfSWIB*^sh-^** | 0.20 | 0.20 | 1.50 | 2.30 | 8.50 |

| **GCA Repeat2** | **0h** | **24h** | **48h** | **72h** | **96h** |
| --- | --- | --- | --- | --- | --- |
| **3D7^sh+^** | 0.20 | 0.20 | 2.10 | 2.80 | 8.70 |
| **3D7^sh-^** | 0.20 | 0.20 | 2.40 | 3.00 | 8.90 |
| ***PfSWIB*^sh+^** | 0.20 | 0.20 | 1.20 | 1.90 | 5.10 |
| ***PfSWIB*^sh-^** | 0.20 | 0.20 | 1.20 | 1.70 | 8.30 |

| **GCA Repeat3** | **0h** | **24h** | **48h** | **72h** | **96h** |
| --- | --- | --- | --- | --- | --- |
| **3D7^sh+^** | 0.20 | 0.20 | 1.10 | 1.40 | 7.40 |
| **3D7^sh-^** | 0.20 | 0.20 | 1.40 | 1.60 | 7.80 |
| ***PfSWIB*^sh+^** | 0.20 | 0.20 | 0.70 | 1.00 | 4.20 |
| ***PfSWIB*^sh-^** | 0.20 | 0.20 | 1.10 | 1.40 | 7.90 |

The initial parasitemia of the four parasite lines were 0.2%. ‘sh+’ denotes shiled 1 induced, while ‘sh-’ denotes shiled 1 not induced. The *PfSWIB*^sh-^ and *PfSWIB*^sh+^ lines denote the same *PfSWIB* clone pre- and post- induced, as well as 3D7^sh+^ and 3D7^sh-^ lines. The parasitemia of each parasite line was counted every 12 h up to 96 h. All assays were performed in triplicate.
