# Supplementary material for: PfSWIB, a potential chromatin regulator for var gene regulation and parasite development in Plasmodium falciparum
Source: Parasit Vectors. 2020 Feb 4;13:48. doi: 10.1186/s13071-020-3918-5 (PMC7001229; doi:10.1186/s13071-020-3918-5)
Supplement: Supplementary file 6 — Additional file 6: Figure S3. RNA-seq analysis of expression profile in different parasite lines. a Comparison of transcriptomes after re-invasion in 3rd life-cycle. b Hierarchical clustering heatmap for all the differentially expressed genes after re-invasion in 3rd life-cycle. [file 13071_2020_3918_MOESM6_ESM.docx]

**
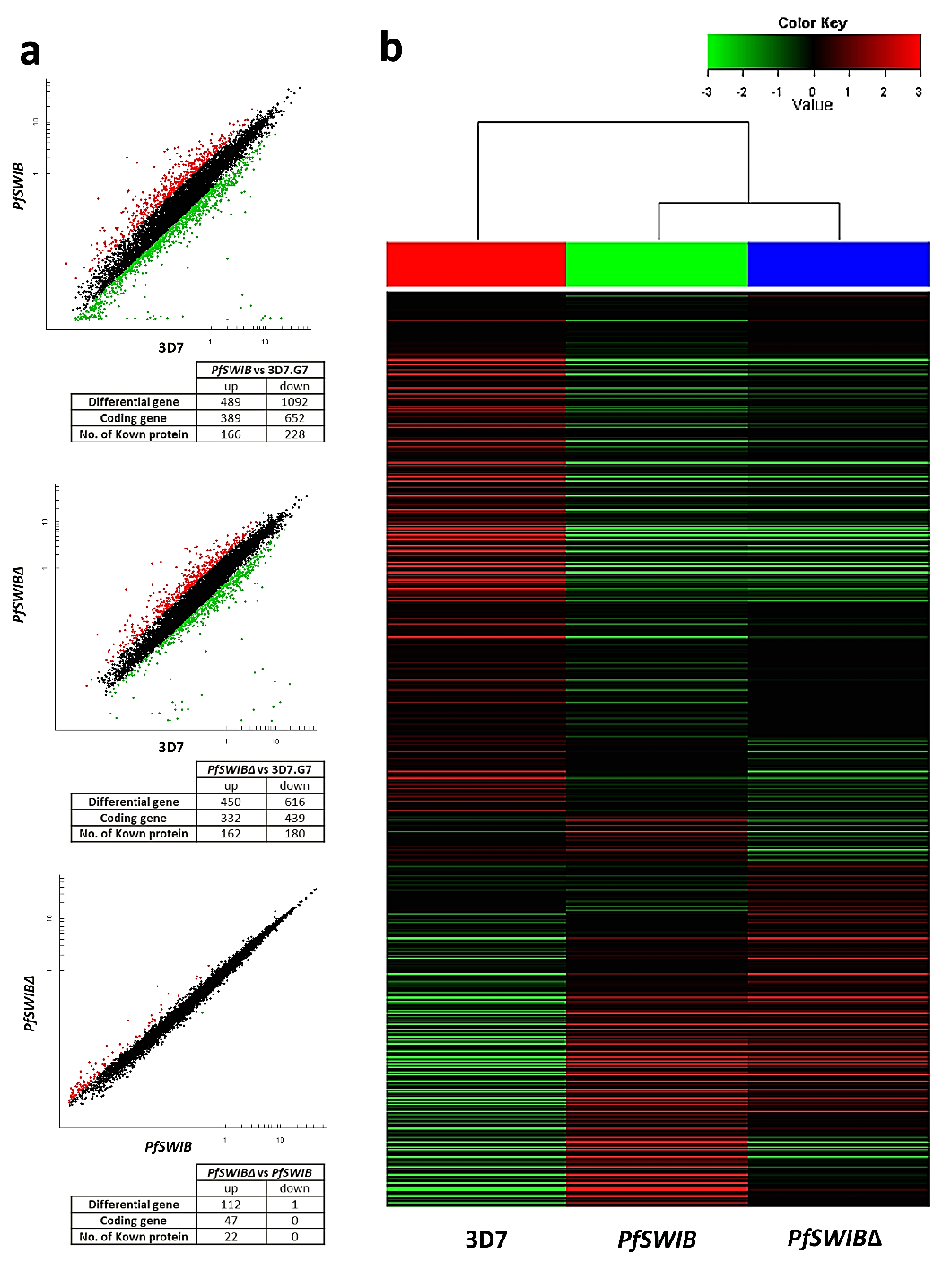
**

**Additional file 6: Figure S3.** RNA-seq analysis of expression profile in different parasite lines. **a** Comparison of transcriptomes of *PfSWIB vs* 3D7, *PfSWIB*∆ *vs* 3D7, and *PfSWIB*∆ *vs PfSWIB* after re-invasion in 3rd life-cycle of *P. falciparum*. X-axis and Y-axis are logarithmic and correspond to relative signal of hybridization to each gene shown as a dot. Red dots denote the upregulated genes (fold change ≥1.5, FDR <0.05, *P* <0.05), while green dots denote the downregulated genes (fold change ≤ -1.5, FDR <0.05, *P* <0.05). **b** Hierarchical clustering heatmap of log_2_-transformed fragments per kilobase of transcript per million (FPKM) gene expression values for all the differentially expressed genes after re-invasion in 3rd life-cycle. The color scale ranges from green to red, showing a range from minimum (≤-0.4) to maximum (≥-0.4) log_2_ FPKM gene expression values for each variant.
