# Supplementary material for: PfSWIB, a potential chromatin regulator for var gene regulation and parasite development in Plasmodium falciparum
Source: Parasit Vectors. 2020 Feb 4;13:48. doi: 10.1186/s13071-020-3918-5 (PMC7001229; doi:10.1186/s13071-020-3918-5)
Supplement: Supplementary file 7 — Additional file 7: Figure S4. Conditional knockdown of PfSWIB leads to a change in mutually exclusive var gene transcription. Data are presented as fold change of var transcription level in the PfSWIB∆ line with respect to the PfSWIB line. [file 13071_2020_3918_MOESM7_ESM.docx]

**
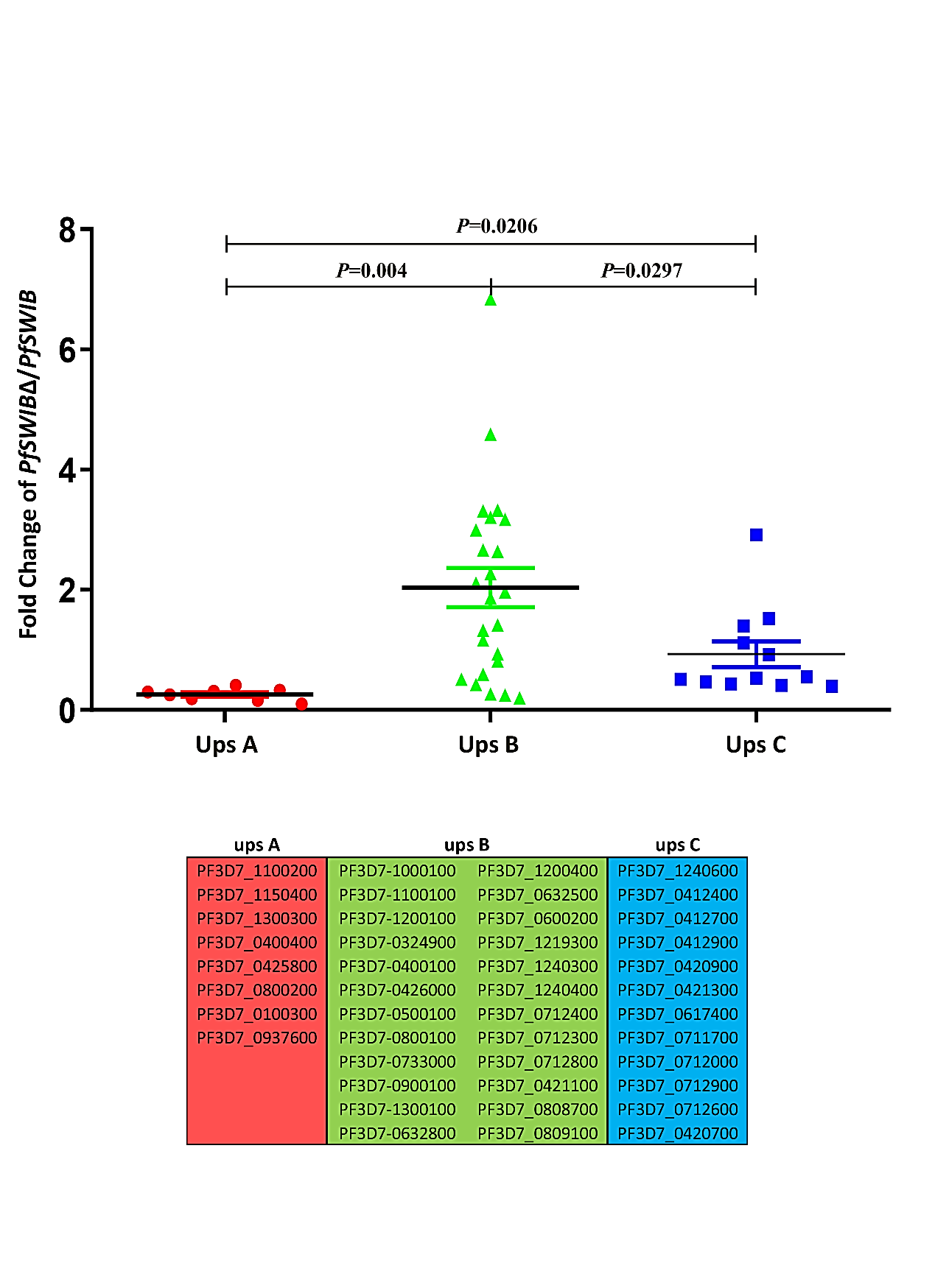
**

**Additional file 7: Figure S4.** Conditional knockdown of *PfSWIB* leads to a change in mutually exclusive *var* gene transcription. Significance and different expression levels of individual *var* genes are demonstrated in scatter plots (GraphPad Prism, version 5.0) in the same clone pre- and post-shield1 induction. Data are presented as fold change of *var* transcription level in the *PfSWIB****∆*** line with respect to the *PfSWIB* line. The error bars represent the mean ± SD of three independent experiments determined by qPCR. The red, green and blue dots indicate the *upsA*-, *upsB*- and *upsC*-subtype *vars*, respectively.
