# Supplementary material for: PfSWIB, a potential chromatin regulator for var gene regulation and parasite development in Plasmodium falciparum
Source: Parasit Vectors. 2020 Feb 4;13:48. doi: 10.1186/s13071-020-3918-5 (PMC7001229; doi:10.1186/s13071-020-3918-5)
Supplement: Supplementary file 8 — Additional file 8: Figure S5. Conditional knockdown of PfSWIB leads to a change in expression profile of 60 vars in different lines. [file 13071_2020_3918_MOESM8_ESM.docx]

**
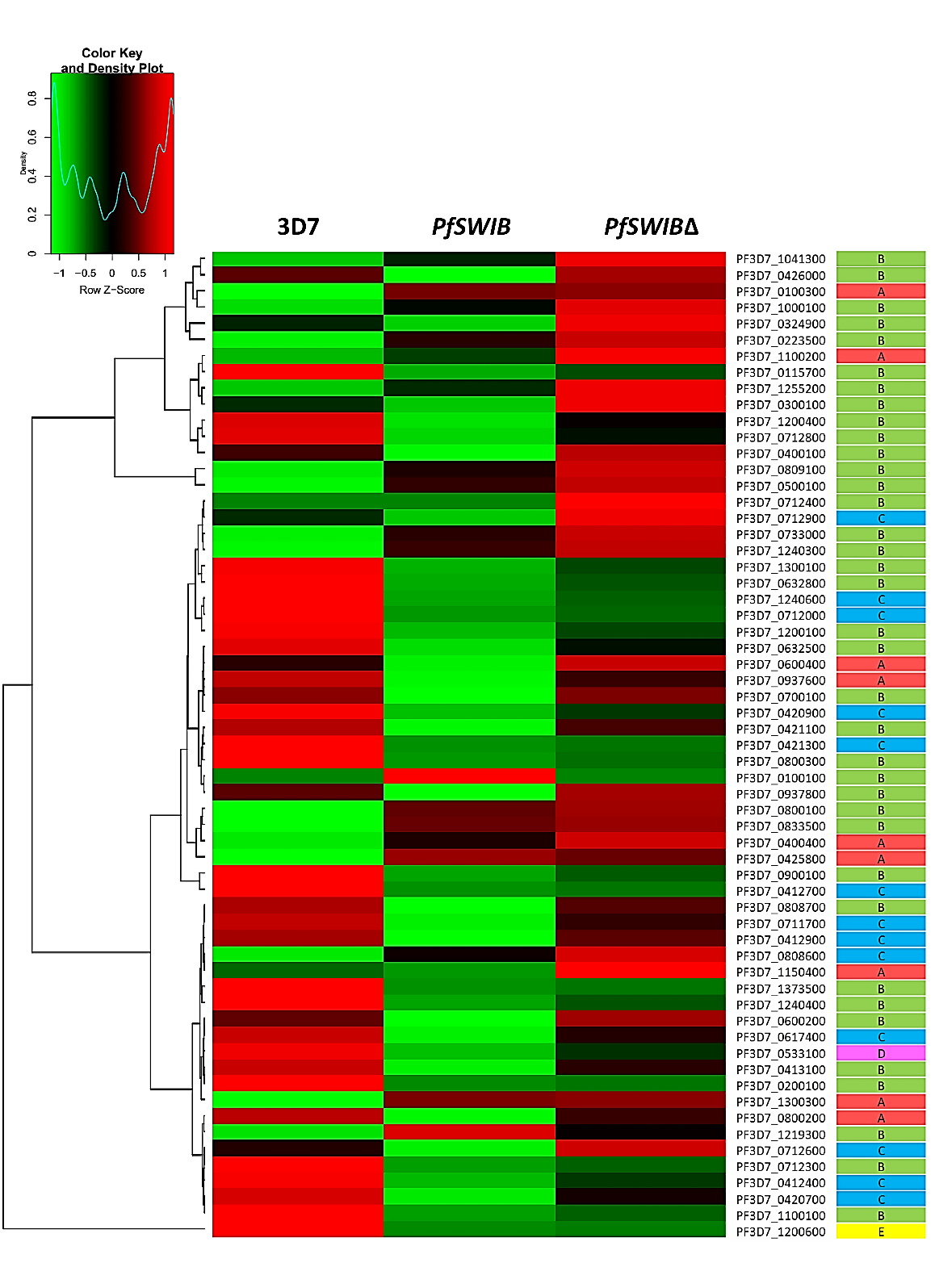
**

**Additional file 8: Figure S5.** Conditional knockdown of *PfSWIB* leads to a change in expression profile of 60 *vars* in different lines. Hierarchical clustering heatmap of log_2_ transformed fragments per kilobase of transcript per million (FPKM) gene expression values for 60 *var* genes after re-invasion in the 3rd life-cycle. The color scale ranges from green to red, showing a range from minimum (≤-0.4) to maximum (≥-0.4) log_2_ FPKM gene expression values for each *var* variant. Different *var* gene subtypes are shown in differently colored boxes.
