# Supplementary material for: PfSWIB, a potential chromatin regulator for var gene regulation and parasite development in Plasmodium falciparum
Source: Parasit Vectors. 2020 Feb 4;13:48. doi: 10.1186/s13071-020-3918-5 (PMC7001229; doi:10.1186/s13071-020-3918-5)
Supplement: Supplementary file 9 — Additional file 9: Figure S6. Linear regression analysis between qPCR and RNA-seq data. Red dots denote the log2 (fold change) of 44 vars in the PfSWIB vs PfSWIB∆ comparison. [file 13071_2020_3918_MOESM9_ESM.docx]

**
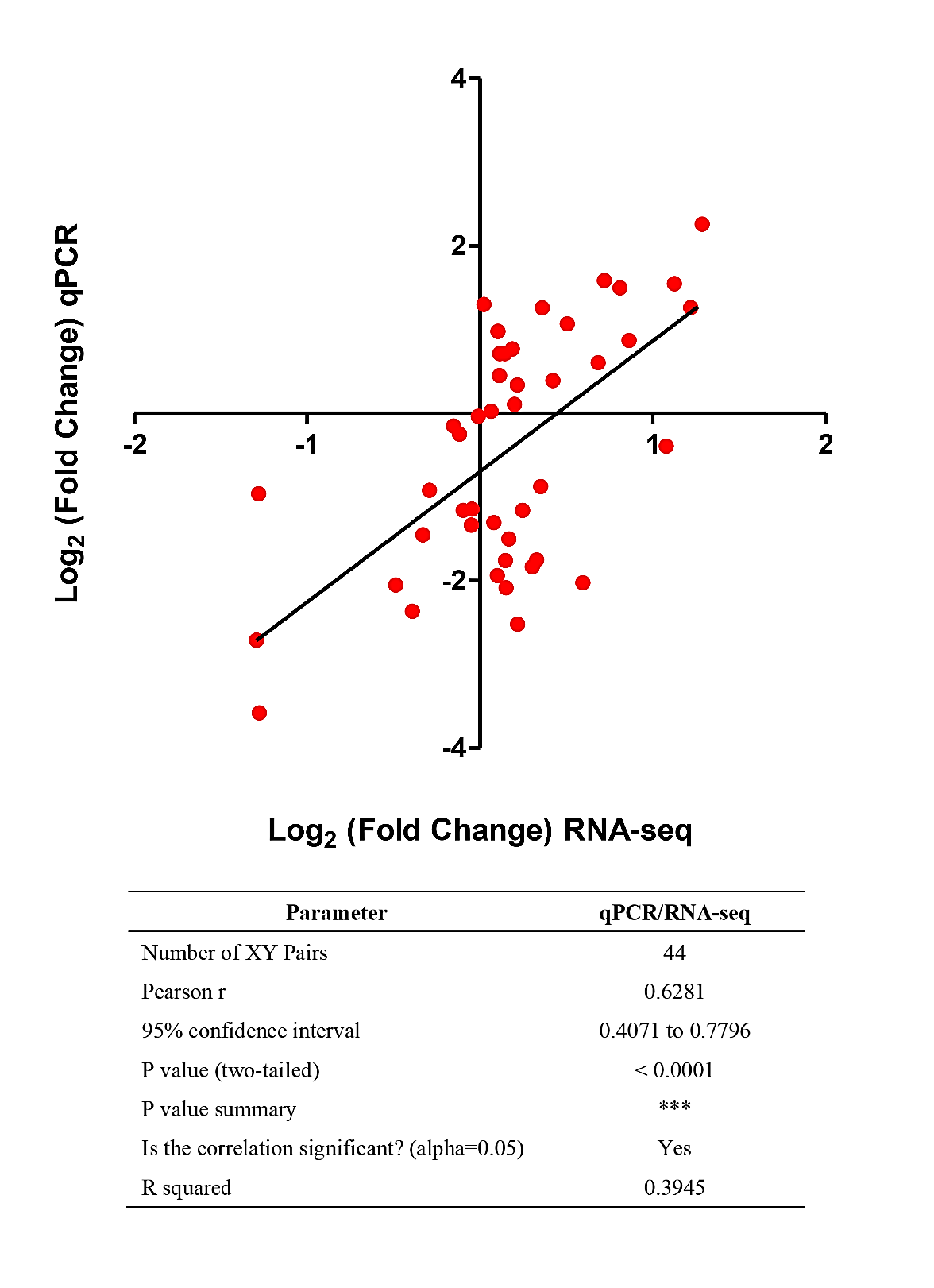
**

**Additional file 9: Figure S6.** Linear regression analysis between qPCR and RNA-seq data. Linear regression analysis of the correlation between qPCR and RNA-seq data was performed to investigate the transcriptome data accuracy on 44 *var* genes. Red dots denote the log_2_ (fold change) of 44 *vars* in the *PfSWIB vs PfSWIB∆* comparison. The linear regression and correlation were analyzed using GraphPad Prism^®^ (version 5.0) and SPSS (version 16.0).
