# Supplementary material for: PfSWIB, a potential chromatin regulator for var gene regulation and parasite development in Plasmodium falciparum
Source: Parasit Vectors. 2020 Feb 4;13:48. doi: 10.1186/s13071-020-3918-5 (PMC7001229; doi:10.1186/s13071-020-3918-5)
Supplement: Supplementary file 10 — Additional file 10: Table S4. Log2 (fold change) qPCR and RNA-seq data of 44 vars in the PfSWIB vs PfSWIB∆ comparison. [file 13071_2020_3918_MOESM10_ESM.docx]

**Additional file 10: Table S4. Log_2_ (Fold change) qPCR and RNA-seq data of 44 *vars* in the *PfSWIB* vs *PfSWIB*∆ comparison.**

| **Gene ID** | **Log_2_(FC) RNA-seq 1** | **Log_2_(FC) RNA-seq 2** | **Log_2_(FC) qPCR 1** | **Log_2_(FC) qPCR 2** |
| --- | --- | --- | --- | --- |
| PF3D7_0100300 | -0.03810371 | 0.33431113 | -1.37000018 | -2.15000010 |
| PF3D7_0324900 | -0.03851980 | 2.19555940 | -0.90000008 | 0.12000018 |
| PF3D7_0400100 | 1.13954576 | 1.43136944 | 0.97000030 | 3.55000055 |
| PF3D7_0400400 | 0.01748774 | 0.41948774 | -3.05000049 | -1.98999993 |
| PF3D7_0412400 | -0.14369952 | -0.51569952 | -1.95999976 | -0.93999995 |
| PF3D7_0412700 | -0.01744330 | -0.21944330 | -0.85000009 | 0.35000042 |
| PF3D7_0412900 | 0.18008766 | -0.01991234 | -1.26999991 | -1.33999984 |
| PF3D7_0420700 | 0.12096894 | 0.72296894 | -0.40999995 | 1.19000036 |
| PF3D7_0420900 | -0.01391061 | -0.56821219 | -1.30999993 | -0.53000007 |
| PF3D7_0421100 | 0.50046806 | -0.09953194 | -1.00000000 | 1.20999977 |
| PF3D7_0421300 | -0.10312948 | -2.45719992 | -1.28000003 | -0.63999988 |
| PF3D7_0425800 | -1.39633643 | -1.19039217 | -3.07000060 | -2.35000018 |
| PF3D7_0426000 | 0.13596306 | 0.58329814 | 0.13000039 | 2.39000011 |
| PF3D7_0500100 | 0.09809188 | 1.52374572 | 0.66000034 | 2.33999989 |
| PF3D7_0600200 | 0.19101510 | 0.51101510 | -1.42999997 | -0.31999996 |
| PF3D7_0617400 | -0.13476582 | 0.79008218 | -3.08000007 | -0.42000005 |
| PF3D7_0632500 | -0.07742563 | -0.02108702 | -0.85999989 | -1.81000015 |
| PF3D7_0632800 | -0.43205002 | 0.12794998 | -0.52000007 | 0.22000051 |
| PF3D7_0711700 | -0.20581814 | 0.69745442 | -1.57999995 | -0.74000013 |
| PF3D7_0712000 | -0.04538237 | 0.02410590 | -0.78999998 | 0.71999997 |
| PF3D7_0712300 | 0.51254501 | 1.73835519 | 0.93000000 | 2.17000002 |
| PF3D7_0712400 | 0.04379890 | 0.08379890 | -0.72000010 | 0.77000018 |
| PF3D7_0712600 | 1.24293410 | 0.48293410 | -0.63000003 | 2.36999991 |
| PF3D7_0712800 | 0.41356418 | 0.01956418 | -0.09000008 | 0.77000018 |
| PF3D7_0712900 | 0.21175332 | 0.01575332 | 0.17000066 | 0.72999992 |
| PF3D7_0733000 | 0.12132144 | 1.31732144 | 0.15999982 | 3.00999993 |
| PF3D7_0800100 | 0.70478117 | 0.30678117 | 0.06000033 | 2.07999994 |
| PF3D7_0800200 | -0.01665430 | 0.34974030 | -2.26999991 | -0.73000003 |
| PF3D7_0808700 | 0.04394910 | 0.24194910 | -0.02000001 | 1.44999973 |
| PF3D7_0809100 | -0.03832027 | 1.40472575 | -0.47000000 | 1.67999977 |
| PF3D7_0900100 | 0.28183699 | 2.15690279 | 0.05000011 | 2.48000009 |
| PF3D7_0937600 | -0.14141296 | 0.44423888 | -1.55999992 | -2.60999978 |
| PF3D7_1000100 | 0.21723774 | 0.15723774 | -0.36999999 | 1.91000000 |
| PF3D7_1100100 | 0.01021834 | 0.21814851 | -0.35000000 | 1.78000011 |
| PF3D7_1100200 | 0.90027810 | 0.28947161 | -2.92999984 | -1.12000008 |
| PF3D7_1150400 | 0.43856620 | 0.16672422 | -2.64999980 | -1.02000015 |
| PF3D7_1200100 | 0.01011444 | 0.03684316 | 0.37000019 | 2.23000006 |
| PF3D7_1200400 | -0.03704495 | -0.15704495 | -1.91000025 | -0.40999995 |
| PF3D7_1219300 | -0.19821416 | -0.77378584 | -2.01999986 | -2.08000007 |
| PF3D7_1240300 | 0.12124760 | 0.08100192 | -2.02999985 | -1.84999983 |
| PF3D7_1240400 | -0.64146662 | -0.14146662 | -2.34000020 | -2.39000001 |
| PF3D7_1240600 | -0.00645242 | -0.08403220 | -0.44000002 | -1.84999983 |
| PF3D7_1300100 | 0.15341454 | 0.05341454 | -0.19000004 | 2.15000004 |
| PF3D7_1300300 | -1.19772942 | -1.35685898 | -4.31000000 | -2.84999983 |

Values in the boxes denote the log_2_ (fold change) of either the RNA-seq or qPCR data. ‘1’ and ‘2’ indicate the qPCR and RNA-seq data selected from two independent experiments.
