# Supplementary material for: PfSWIB, a potential chromatin regulator for var gene regulation and parasite development in Plasmodium falciparum
Source: Parasit Vectors. 2020 Feb 4;13:48. doi: 10.1186/s13071-020-3918-5 (PMC7001229; doi:10.1186/s13071-020-3918-5)
Supplement: Supplementary file 11 — Additional file 11: Figure S7. Detection of Var gene expression pattern during a single 48-hour life-cycle in P. falciparum clone 3D7. Abbreviations: R, ring; T, trophozoite; Key: +, shield1 induced; -, shield1 not induced. [file 13071_2020_3918_MOESM11_ESM.docx]

**
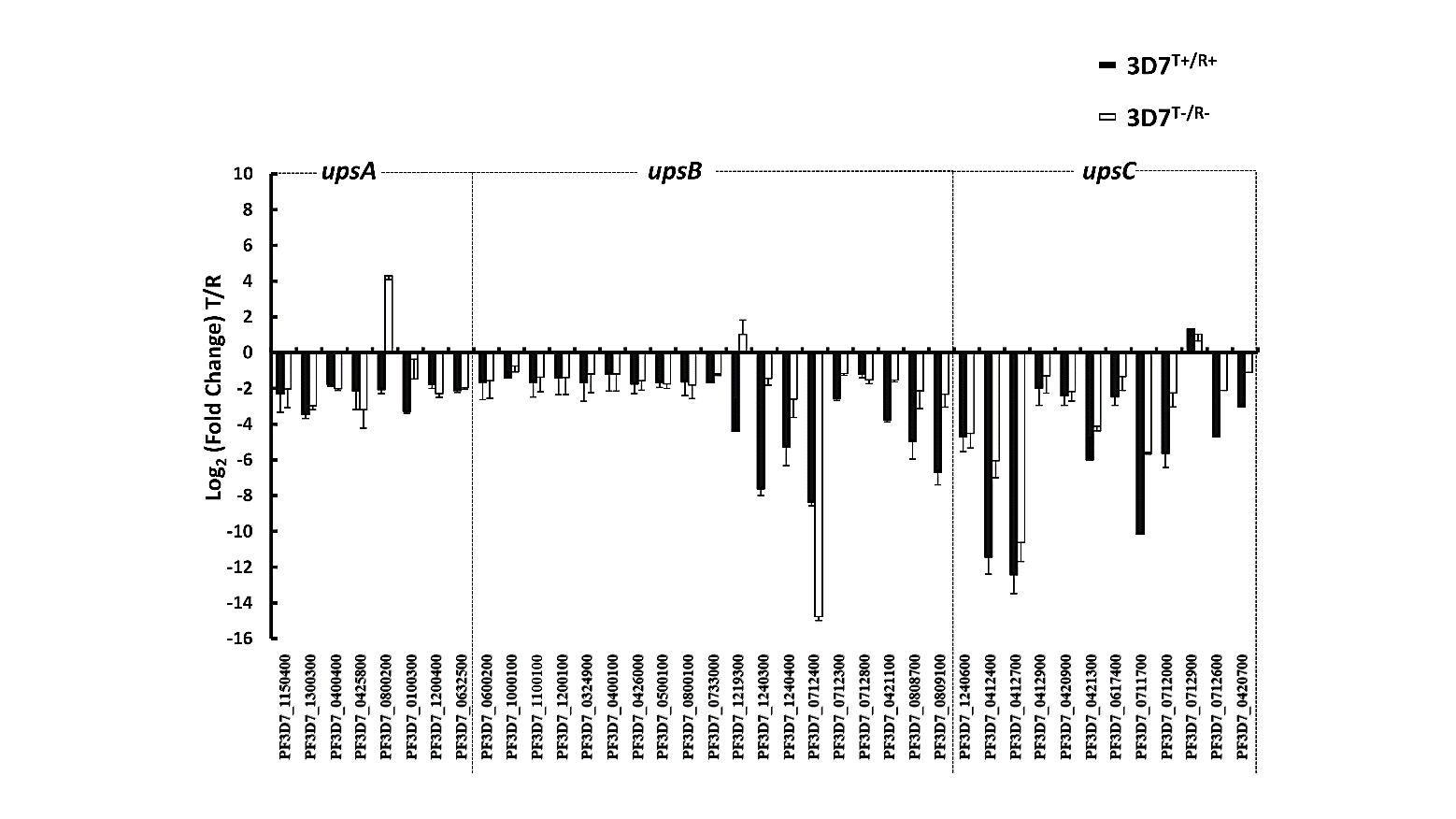
**

**Additional file 11: Figure S7.** Detection of *Var* gene expression pattern during a single 48-hour life-cycle in *P. falciparum* clone 3D7. qPCR analysis of the transcription level of individual *var* genes in 3D7 clone during a single 48-hour life-cycle of *P. falciparum*. The data are presented as log_2_ fold change of trophozoite to ring within the third life-cycle, using the seryl-tRNA synthetase gene (GenBank: PF3D7_0717700) as an endogenous control. The error bars represent the mean ± SD of three independent experiments determined by qPCR. *Abbreviations*: R, ring; T, trophozoite; *Key*: +, shield1 induced; -, shield1 not induced.
